# Claspin haploinsufficiency leads to defects in fertility, hyperplasia and an increased oncogenic potential

**DOI:** 10.1042/BCJ20220101

**Published:** 2022-10-14

**Authors:** Suzanne Madgwick, Saimir Luli, Helene Sellier, Jacqueline A. Butterworth, Jack Leslie, Adam J. Moore, Emma K. Corbin, Adrian I. Yemm, Robson T. Chiremba, Dina Tiniakos, Fiona Oakley, Neil D. Perkins, Jill E. Hunter

**Affiliations:** 1 Newcastle University Biosciences Institute, Wolfson Childhood Cancer Research Centre, Level 6, Herschel Building, Newcastle University, Brewery Lane, Newcastle upon Tyne NE1 7RU, U.K.; 2 Preclinical In Vivo Imaging Facility, Faculty of Medical Sciences, Newcastle University, Newcastle Upon Tyne NE2 4HH, U.K.; 3 Newcastle Fibrosis Research Group, Newcastle University Biosciences Institute, Faculty of Medical Sciences, Newcastle University, Newcastle Upon Tyne NE2 4HH, U.K.

**Keywords:** Clapsin, DNA replication and recombination, genome integrity, hepatocellular carcinoma, hyperplasia, oocyte

## Abstract

Claspin is an adaptor protein required for ATR-dependent phosphorylation of CHK1 during S-phase following DNA replication stress. Claspin expression is highly variable in cancer, with low levels frequently correlating with poor patient survival. To learn more about the biological consequences of reduced Claspin expression and its effects on tumorigenesis, we investigated mice with a heterozygous knockout of the *Clspn* gene. Claspin haploinsufficiency resulted in reduced female fertility and a maternally inherited defect in oocyte meiosis I cell cycle progression. Furthermore, aged *Clspn^+/−^* mice developed spontaneous lymphoid hyperplasia and increased susceptibility to non-alcoholic fatty liver disease. Importantly, we demonstrate a tumour suppressor role for Claspin. Reduced Claspin levels result in increased liver damage and tumourigenesis in the DEN model of hepatocellular carcinoma. These data reveal that *Clspn* haploinsufficiency has widespread unanticipated biological effects and establishes the importance of Claspin as a regulatory node controlling tumorigenesis and multiple disease aetiologies.

## Introduction

DNA replication stress results from stalled DNA replication forks and is a feature of cancer cell biology leading to genomic instability, a driver of clonal evolution [1–3]. Replication stress can be induced by a variety of mechanisms including DNA damaging agents, and by oncoproteins such as MYC driving hyper-DNA replication [1–3]. Critical regulators of the cellular response to DNA replication stress are the checkpoint kinases ataxia telangiectasia and Rad3 related (ATR) and checkpoint kinase 1 (CHK1), which protect against tumorigenesis by promoting DNA repair or apoptosis [3,4]. However, once established, tumour cells can also become addicted to this pathway since it enables them to survive ongoing, potentially lethal, genomic instability [5].

Many proteins are known to regulate the DNA replication stress checkpoint response, these include the co-regulators Claspin (encoded by the *CLSPN* gene), ATRIP, TopBP1 and Rad17 that are all required for ATR-mediated activation of CHK1 [6,7]. In S-phase Claspin is also required for the safeguarding and monitoring of genome duplication, is fundamental in maintaining replication rates, and is found within the replisome where it regulates origin firing [7]. Claspin is, therefore, an essential mediator of the DNA damage-induced S-phase checkpoint.

Claspin's function as a regulator of genomic stability suggests a potential role during tumorigenesis [8] though there is no direct *in vivo* evidence for this. Many studies have documented increased levels of Claspin in tumour *versus* normal tissue [8,9], or correlated an increase in Claspin expression with malignant progression [10,11]. However, these reports analyse established tumour tissue only and it may be postulated that these tumours have up-regulated Claspin to cope with a high replicative index. Importantly, studies investigating the functional role of Claspin in the onset and progression of tumourigenesis are absent, there is little data relating Claspin function to other disease states and, a role for Claspin in aging tissue is unknown. Here, for the first time, we examine the role of Claspin *in vivo* using a colony of *Clspn^+/−^* mice revealing profound, widespread and unexpected biological effects of Claspin haploinsufficiency.

## Results

### Claspin mRNA levels predict overall survival in Eμ-Myc B-cell lymphoma and correlate with survival times in human cancers

**Figure 1.**
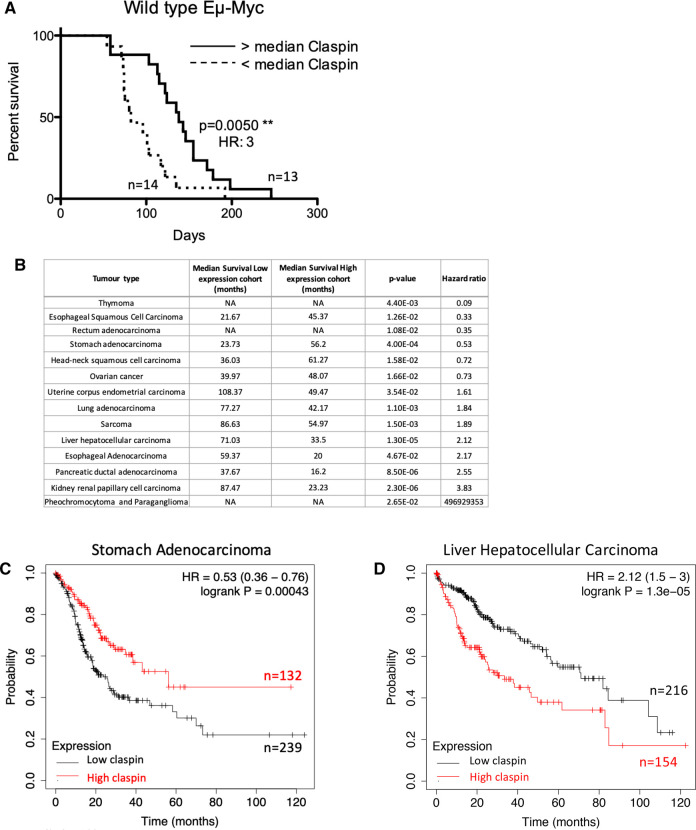
Low Claspin levels are associated with poor outcome in Eμ-Myc lymphomas and human stomach adenocarcinoma. (**A**) WT Eμ-Myc mice with lower Claspin levels have reduced overall survival. Kaplan–Meier survival analysis of WT Eμ-Myc mice comparing above (*n* = 13) and below (*n* = 14) median level expression of *Clspn* mRNA. Overall survival is significantly shorter in Eμ-Myc mice with low levels of Claspin transcript (**P = 0.005Mantel–Cox test and hazard ratio (HR) analysis indicate that these mice are at 3 times greater risk of dying earlier due to lymphoma compared with Eμ-Myc mice with high levels of Claspin transcript. Please note that this plot is also used in another study [12], Figure 8C. (**B**) Table summarising Kaplan–Meier plot analysis to determine the correlation of CLSPN expression levels with human cancer patient survival. Analysis was performed using the pan-cancer RNA Seq database at kmplot.com [15]. Data presented is for cancer types where the logrank *P*-value was <0.05. Please note that a score of NA is given when either the low or high Clspn cohort (or both) does not drop below a probability of survival of 0.5. See also Supplementary Data File S1. (**C**,**D**) Graphs depicting examples from the Kaplan–Meier plot analysis described in Figure 1B that (**C**) show the correlation of low CLSPN expression levels with poor survival in stomach cancer patients. Patients with low *CLSPN* mRNA levels (black line *n* = 239) have significantly shorter overall survival (*P* = 0.00043 Mantel–Cox test, HR 0.53) compared with patients with high Claspin expression (red line *n* = 132), and (**D**) show the correlation of high CLSPN expression levels with poor survival in liver hepatocellular carcinoma cancer patients. In this case, patients with low *CLSPN* mRNA levels (black line *n* = 216) have significantly longer overall survival (*P* = 0.000013 Mantel–Cox test, HR 2.12) compared with patients with high Claspin expression (red line *n* = 154).

Parallel studies using the Eμ-Myc mouse model of B-cell lymphoma demonstrated that low levels of *Clspn* mRNA were associated with significantly reduced overall survival times. Mice with low levels of *Clspn* transcript had a median survival of 82 days compared with 138 days for animals expressing higher levels of *Clspn* in this model (Figure 1A and [12]). This suggests that high levels of Claspin are protective and delay onset of disease in this particular mouse model. These data suggest that a failure to induce Claspin expression in tumour cells leads to a defective oncogene-induced DNA damage response and increased genomic instability, likely contributing to the earlier onset of disease. We, therefore, next investigated whether *CLSPN* mRNA levels are also predictive of cancer progression in humans. Using publicly available datasets on KM Plotter [13] and Human Protein Atlas (www.proteinatlas.org) [14], we determined the prognostic implications of Claspin expression in many different types of human cancer.

Strikingly, analysis of CLSPN expression in different cancer cell types using patient RNA Seq data from KM Plotter [15] revealed that, similar to the Eμ-Myc mouse model (Figure 1A and [12]), low levels of *CLSPN* transcripts can be significantly associated with worse patient survival (Figure 1B,C and Supplementary Data File S1) [15,16]. Moreover, The Human Protein Atlas indicates that for stomach cancer (Figure 1C), low levels of Claspin expression are a prognostic marker for worse survival (https://www.proteinatlas.org/ENSG00000092853-CLSPN/pathology/stomach+cancer). However, this was not the case in all tumour types and in some cases, such as liver hepatocellular carcinoma (HCC) and pancreatic cancer, the opposite effect was observed (Figure 1B,D and Supplementary Data File S1). This indicates a more complex situation in human cancers. Note that this analysis only takes into account expression levels in developed tumours, the association of high CLSPN levels with poor prognosis may reflect a situation where this pathway is used to cope with high levels of DNA replication stress in some cancers. Nonetheless, these data suggest that Claspin levels can be a prognostic cancer indicator and importantly could influence the response to therapy, particularly of checkpoint kinase inhibitors [5], in human patients.

### Female *Clspn*^+/−^ mice have lower litter numbers and fewer successful pregnancies

To resolve whether reduced Claspin levels alone can be sufficient to enhance tumorigenesis we studied *Clspn* knockout mice developed by the KOMP. In these studies, heterozygous knockout *Clspn* mice were used since, similar to other genes associated with DNA replication stress, homozygous loss of the *Clspn* gene is embryonic lethal at E10.5 (our observation and http://www.mousephenotype.org/data/genes/MGI:2445153).

**Figure 2.**
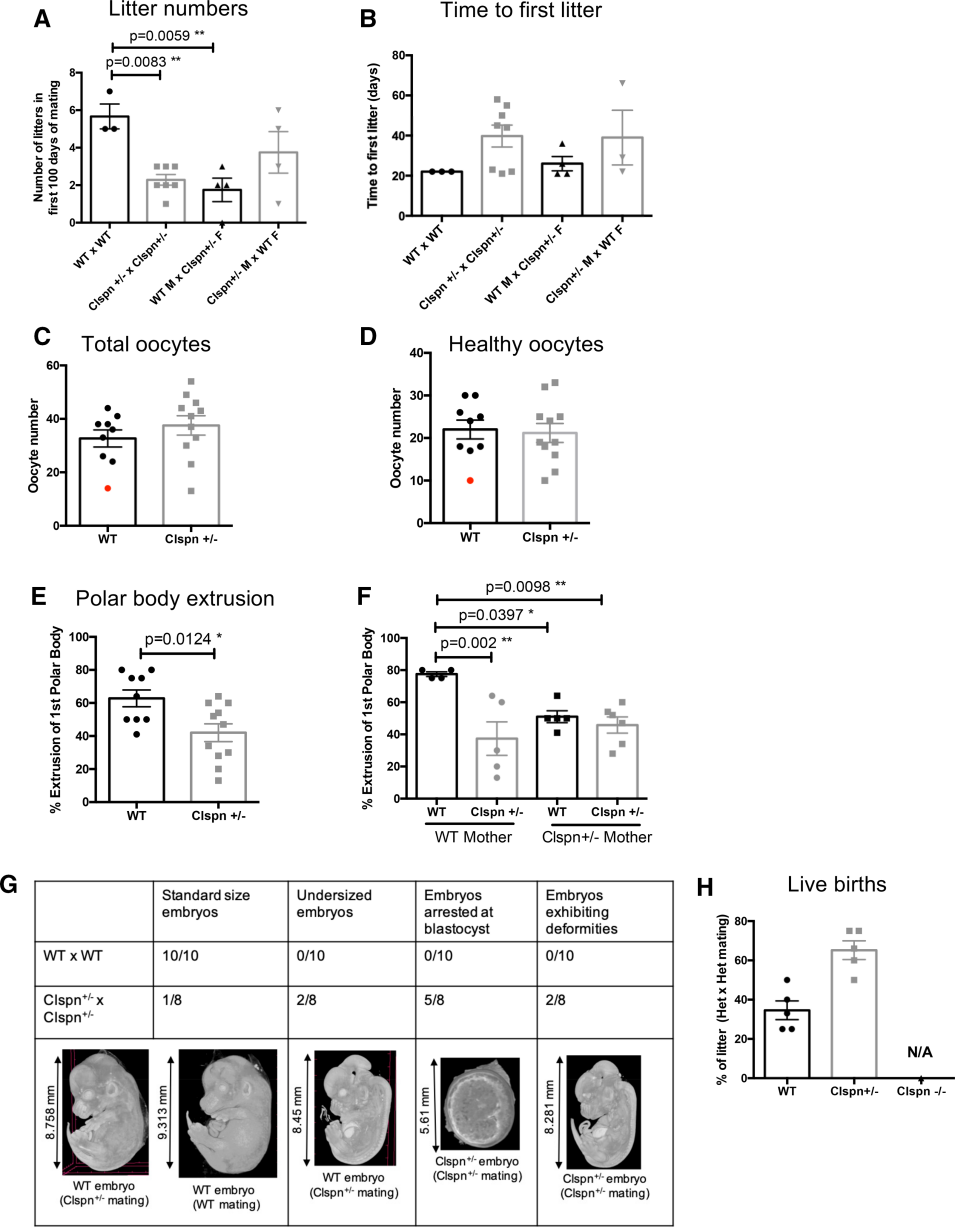
*Clspn^+/−^* mice have a reduced reproductive capacity. (**A**) Scatter plot showing the number of viable litters born in the first 100 days of mating in a range of different matings; WT male and WT females (*n* = 3), *Clspn^+/−^* male and *Clspn^+/−^* females (*n* = 7), WT male and *Clspn^+/−^* females (*n* = 4) or *Clspn^+/−^* male and WT females (*n* = 4). Analysed using an one-way ANOVA with Sidak *post-hoc* test. (**B**) Scatter plot showing the number of days to the first viable litter born in a range of different matings; WT male and WT females (*n* = 3), *Clspn^+/−^* male and *Clspn^+/−^* females (*n* = 7), WT male and *Clspn^+/−^* females (*n* = 4) or *Clspn^+/−^* male and WT females (*n* = 3). Analysed using an one-way ANOVAa with Sidak *post-hoc* test. (**C**) Scatter plot showing the total number of oocytes in 6-week-old WT (*n* = 9) and *Clspn^+/−^* females (*n* = 11). The red dot indicates a mouse with only one ovary. Unpaired Student's *t*-test were performed but no significant differences were detected. (**D**) Scatter plot showing the number of healthy oocytes in 6-week-old WT (*n* = 9) and *Clspn^+/−^* females (*n* = 11). The red dot indicates a mouse with only one ovary. Unpaired Student's *t*-test were performed but no significant differences were detected. (**E**,**F**) *Clspn^+/−^* oocytes cannot efficiently exit meiosis I. (**E**) Scatter plot showing that *Clspn^+/−^* oocytes fail to extrude the first polar body required to progress through meiosis. WT oocytes (*n* = 8) can more efficiently extrude the first polar body than *Clspn^+/−^* oocytes (*n* = 11) (*P = 0.0126 Unpaired student's *t*-test). (**F**) Scatter plots showing that maternal genotype can impact on offspring fertility; all *Clspn^+/−^* oocytes have significantly reduced ability to extrude the first polar body. WT female mice born from *Clspn^+/−^* mothers (*n* = 4) have significantly reduced ability to extrude the first polar body compared with WT females born from WT mothers (*n* = 4). Analysed using an one-way ANOVA with Sidak *post-hoc* test. (**G**) Embryos from *Clspn^+/−^* matings exhibit a range of abnormalities at E13.5, ranging from undersized pups to arrest at blastocyst stage. Representative images are shown using computerised tomographic images at each developmental stage. (**H**) Scatter plot showing the percentage of live births that are WT (*n* = 5) or *Clspn^+/−^* (*n* = 5) in litters from *Clspn^+/−^* males mated with *Clspn^+/−^* females. No *Clspn^−/−^* pups were born due to the embryonic lethality observed.

While establishing the colony of *Clspn^+/−^* mice, a number of phenotypes emerged to demonstrate the clear, detrimental effect of Claspin haploinsufficiency. Strikingly, Claspin haploinsufficiency had a dramatic effect on female reproductive function; matings containing *Clspn^+/−^* female mice had significantly fewer litters (2.2 compared with 5 for wild-type (WT) mice in 100 days; Figure 2A), lengthened time to first litter (44.5 days compared with 22 days for WT mice; Figure 2B) and a reduced average number of pups per litter (3.8 compared with 7.2 for WT; Supplementary Figure S1A), indicative of substantially reduced reproductive capacity in *Clspn^+/−^* female mice. There was no significant difference in ovary weight from *Clspn^+/−^* and WT female mice (Supplementary Figure S1B,C) but we were able to collect a slightly increased number of total oocytes from *Clspn^+/−^* mice (Figure 2C). The number of apparently healthy oocytes (Figure 2D) from *Clspn^+/−^* and WT female littermates at 6 weeks of age were similar but, interestingly we recovered more oocytes that were classified as either immature, or had failed to arrest at the correct stage of their cell cycle from *Clspn^+/−^* females (though this was not significant with our n numbers; Supplementary Figure S1D–G).

Following this initial scoring for morphological features of oocyte health, all oocytes were released from prophase I arrest and cultured to support their maturation through meiosis I (MI) cell division. In both WT and *Clspn^+/−^* mice, >90% of all ‘immature’ or ‘unhealthy’ oocytes either failed to resume meiosis or, failed to establish an aligned meiosis II (MII) spindle on completion of MI. This observation was expected and confirms that we have correctly identified those oocytes incapable of being fertilised. Furthermore, where ‘healthy’ oocytes were allowed to mature, there was no difference in the rate of meiotic resumption between WT and *Clspn^+/−^* oocytes, in each experimental replicate 80–90% of oocytes underwent Germinal Vesicle Break Down with normal timings regardless of mouse genotype (data not shown).

In contrast, significantly more oocytes from WT mice completed MI than from *Clspn^+/−^* mice (as indicated by the rate of 1st polar body; PB1 extrusion, Figure 2E). Oocytes that failed to extrude a PB1 instead arrested prematurely between mid-late prometaphase I, and did not progress to the correct MII stage necessary for sperm-triggered cell cycle resumption and fertilisation [17]. We suggest that prematurely arrested oocytes from *Clspn^+/−^* females fail to satisfy a critical checkpoint in MI, likely as a result of an impaired ability to repair DNA damage [18]. These data together explain why *Clspn^+/−^* females produce fewer pups per litter and, at least partially explain why they also have fewer litters.

However, though our finding above was significant, we were concerned by the broad range of PB1 extrusion rates in WT mice since we expect little variation and a minimum 75% PB1 extrusion in WT populations under our strict culture conditions [19]. To investigate this, we reanalysed our data, not only based on the genotype of the harvested mouse, but by the genotype of her mother (either WT or *Clspn^+/−^*). It is reasonable to suggest the mothers’ genotype could have a significant effect on her daughter's fertility since all oocytes develop *in utero* [20]. In the case of a WT mouse developing in the uterine environment of a *Clspn^+/−^* female, though genotypically WT, initial embryonic cycles will take place with Claspin haploinsufficiency before maternal mRNA stores are depleted and the embryo begins its own programme of gene transcription. Importantly, it has also been demonstrated that MI and MII oocytes, and preimplantation embryos contain complex patterns of 3D interactions between genes, promoters and enhancers, consistent with their potential to encode maternal epigenetic memory; the inheritance of ‘transcriptional states’ passed on from the primordial germ cell to the embryo [21]. Indeed, reanalysis of PB1 extrusion rates revealed that the oocytes from WT female mice but with a *Clspn^+/−^* mother also had a defect in MI, with significantly reduced extrusion of the polar body comparable to their *Clspn^+/−^* littermates. This remarkable result demonstrates a clear influence of maternal Claspin haploinsufficiency on the fertility of daughter mice, regardless of genotype (Figure 2F).

Given the range of oocyte defects (Figure 2E,F and Supplementary Figure S1D–F), we wanted to further investigate the consequences of Claspin haploinsufficiency on the ability of females to produce young. Therefore, we set up timed matings between WT males and females, and *Clspn^+/−^* males and females, and used computerised tomography to establish the differences between WT and *Clspn^+/−^* embryos. At E13.5, WT matings resulted in 10/10 embryos of the expected size and maturation [22]; (Figure 2G and Supplementary Figure S1H) while *Clspn^+/−^* matings resulted in a range of embryonic developmental stages. Regardless of their genotype (WT or *Clspn^+/−^*), the majority of embryos (7/8) produced by *Clspn^+/−^* matings were smaller than embryos produced by WT matings (Figure 2G). Interestingly, only one WT offspring from *Clspn^+/−^* matings appeared to develop normally. In contrast, *Clspn^+/−^* embryos presented with substantial developmental defects ranging from stalling as blastocysts (5/8) to limb deformities (2/8), suggesting that these pregnancies would not produce viable young (Figure 2G). Furthermore, we found evidence of pregnancy re-absorption in *Clspn^+/−^* matings at E17.5 (data not shown). These matings were all first-time pregnancies, and as such indicate a high first pregnancy failure rate in the *Clspn^+/−^* matings, supporting the lengthened time to first litter we have observed (Figure 2B). Our data suggested that this improves over time and that *Clspn^+/−^* females do go on to have full-term pregnancies and produce healthy offspring which are born at expected Mendelian ratios, albeit in the absence of *Clspn^−/−^* pups (Figure 2H). Taken together, these data confirmed that Claspin haploinsufficiency has a significant impact on biological function and so we proceeded with further analysis of the *Clspn^+/−^* mice.

### Aged *Clspn*^+/−^ mice develop spontaneous lymphoid hyperplasia

**Figure 3.**
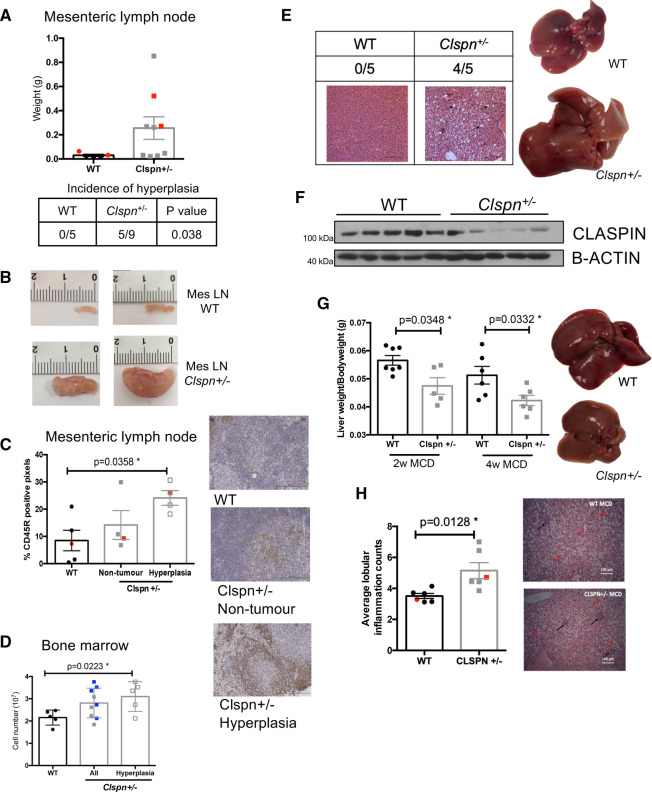
Low Claspin levels lead to spontaneous B-cell lymphoid hyperplasia and non-alcoholic fatty liver disease in aged *Clspn^+/−^* mice. (**A**,**B**) 18-month-old *Clspn^+/−^* spontaneously develop hyperplasia of the mesenteric lymph node. (**A**) Weight of the mesenteric lymph nodes from 18-month-old WT (*n* = 5) and *Clspn^+/−^* mice (*n* = 9). *Clspn^+/−^* mice are more likely to develop hyperplasia (*P = 0.038 *χ*^2^ analysis). (**B**) Representative images of the mesenteric lymph node from two 18-month-old WT and two 18-month-old WT *Clspn^+/−^* mice. Red dots in scatter plot (**A**) indicate samples used in (**B**). (**C**) Scatter plots showing an increase in staining of the lymphocyte marker, CD45R in lymph nodes from *Clspn^+/−^* mice with hyperplasia (*n* = 4), with representative images. Red dots in the scatter plot in indicate sample images used. *Clspn^+/−^* ‘non-tumour’ mice are those that did not exhibit any obvious signs of hyperplasia at the time of harvest. Analysed using an one-way ANOVA with Sidak *post-hoc* test. (**D**) Scatter plot showing an increased bone marrow cellularity in aged *Clspn^+/−^* mice. The middle bar includes ‘All’ of the *Clspn^+/−^* mice but the ones in which hyperplasia was observed are indicated with blue dots in the scatter plot. There is also a bar showing only *Clspn^+/−^* mice which develop hyperplasia. Analysed using an one-way ANOVA with Sidak *post-hoc* test. (**E**) Table showing the incidence of steatohepatitis in WT and *Clspn^+/−^* mice and representative in H&E stained livers of WT and *Clspn^+/−^* mice. Black arrows indicate fat droplets and white arrows indicate inflammatory infiltrates, both higher the *Clspn^+/−^* mice. (**F**) Western blot analysis of Claspin levels in five livers from WT and *Clspn^+/−^* mice. (**G**) *Clspn^+/−^* mice develop NASH earlier after being fed MCD diet. Scatter plots and representative images showing a significant decrease in the liver: bodyweight ratio of *Clspn^+/−^* mice after MCD exposure. Two-week MCD WT (*n* = 6), *Clspn^+/−^* (*n* = 5), 4-week MCD WT (*n* = 6), *Clspn^+/−^* (*n* = 6). Analysed using an one-way ANOVA with Sidak *post-hoc* test. (**H**) NASH is more advanced in *Clspn^+/−^* mice after MCD feeding. Scatter plots showing an increase in lobular inflammation in *Clspn^+/−^* mice (*n* = 6) compared with WT mice (*n* = 6). Red dots in scatter plot in indicate sample images used. H&E stained livers of WT and *Clspn^+/−^* mice. Black arrows indicate the location of lobular inflammation and red arrows indicate fat droplets, both higher the *Clspn^+/−^* mice. Analysed using an Unpaired student's *t*-test.

To investigate the consequences of reduced levels of Clspn, we aged *Clspn^+/−^* mice, the offspring of *Clspn^+/−^* fathers and WT mothers. WT mothers were used to ensure sufficient numbers were generated per breeding pair and, to avoid introducing an uncontrolled variable (namely that genetically WT daughters from *Clspn^+/−^* mothers are not phenotypically normal) and we cannot rule out that difference may extend beyond their ability to reproduce. We observed hyperplasia of the mesenteric lymph node in five out of nine 18-month-old *Clspn^+/−^* mice analysed, but not in any of the age-matched WT littermates (Figure 3A,B). In some *Clspn^+/−^* mice there was also additional hyperplasia in other lymph nodes (2/9) and of the small intestine/colon (2/9) (Supplementary Figure S2A). Immunohistochemical analysis showed that mesenteric lymph nodes exhibiting this hyperplasia stained positive for the lymphocyte marker, CD45R (Figure 3C), suggesting this result was driven by increased lymphocyte proliferation *in situ*. Moreover, aged *Clspn^+/−^* mice that exhibited mesenteric lymph node hyperplasia also had an increased bone marrow cellularity (Figure 3D). At all timepoints at which *Clspn^+/−^* animals were harvested, the mesenteric lymph nodes appeared larger, however, this was not statistically significant (Supplementary Figure S2B,C). No overall differences were seen with other lymph nodes (Supplementary Figure S2D–I). Consistent with our data from the Eμ-Myc model [12], these data suggest that reduced Claspin expression alone results in increased lymphoid hyperplasia that could contribute to the onset of lymphoma.

### *Clspn^+/^*^−^ mice are susceptible to non-alcoholic fatty liver disease

The aged colony of *Clspn^+/−^* mice exhibited another striking phenotype; 12-month-aged male, but not female, *Clspn^+/−^* mice gained weight more rapidly than WT counterparts (Supplementary Figure S3A,B) and had both heavier livers and a higher liver to body weight ratio than aged-matched WT littermates (Supplementary Figure S3C). At 12 months of age, the livers of four of the five *Clspn^+/−^* mice were exhibiting macroscopic signs of damage, had large, widespread fat droplets and showed inflammatory infiltrates that were indicative of both micro- and macro-steatohepatitis (Figure 3E), in contrast, this was not seen in any WT littermate. Western blot analysis confirmed that Claspin protein levels were reduced in the *Clspn^+/−^* livers (Figure 3F).

Non-alcoholic steatohepatitis [23] is a serious form of non-alcoholic fatty liver disease (NAFLD), characterised by a build-up of fatty deposits in the liver, thought to be a consequence of a sedentary lifestyle coupled with a high calorific intake. This leads to inflammation, liver damage and increased risk of developing HCC [24,25]. Little is known about the role of Claspin, or other checkpoint proteins, in non-alcoholic steatohepatitis (NASH). To investigate this, we took advantage of the methionine and choline deficient [26] diet. Methionine and choline are indispensable for hepatic very-low-density lipoprotein (VLDL) synthesis and mitochondrial β-oxidation. Coupling this to a high sucrose (40%) and enriched fat content (10–20%) [27,28], the MCD diet is a very reproducible method of steatosis-induced liver disease in rodents. Following very short periods on the MCD diet, *Clspn^+/−^* mice responded poorly, losing significant liver weight (Figure 3G) while also displaying increased lobular inflammation (Figure 3H). The latter is indicative of a more advanced disease state in both rodents and humans [29,30]. Taken together these data suggest that Clspn haploinsufficiency results in an increased proportion of damaged hepatocytes, which coupled with the increased fat deposits, induce inflammation and ultimately steatosis and/or HCC.

### Claspin is a regulator of hepatocyte proliferation following partial hepatectomy

The data from aged *Clspn^+/−^* mice and the Eμ-Myc model suggested that Claspin haploinsufficiency could lead to earlier onset of tumorigenesis (Figure 3A–C). Moreover, our analysis of aged *Clspn^+/−^* mice demonstrated an important role for Claspin in the liver (Figure 3E–H). However, high Claspin mRNA levels correlate with poor survival in human cancer (Figure 1A), although these data reflect the situation in end-stage tumours rather than any role for Claspin in the earlier stages of tumorigenesis. Therefore, we decided to investigate this further by investigating Claspin function *in vivo* using models that require hepatocyte proliferation and DNA replication, where it could be expected that Claspin might function as a regulator of proliferation and tumorigenesis.

**Figure 4.**
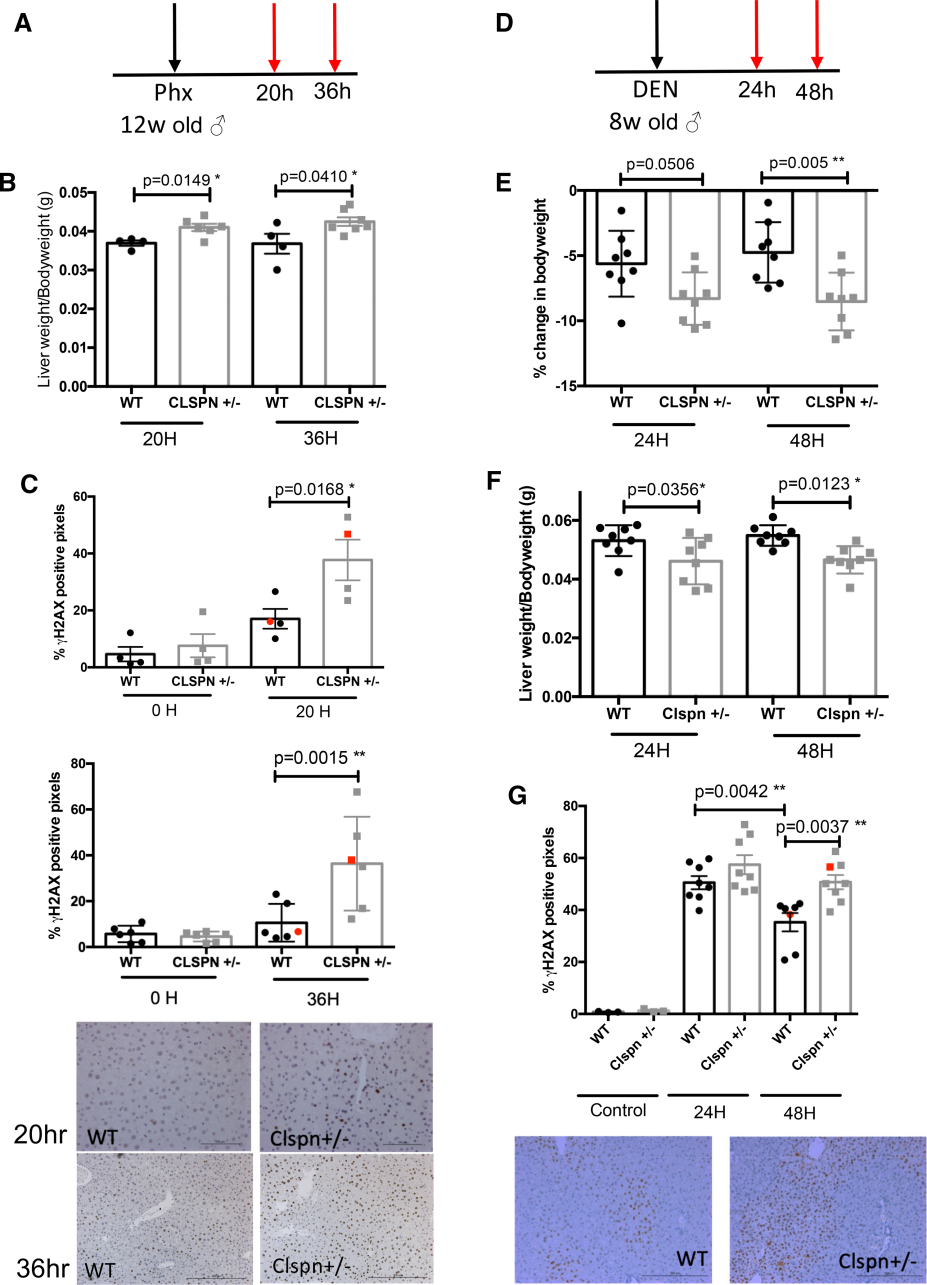
*Clspn^+/−^* mice are more susceptible to acute liver injury. (**A**) Schematic diagram illustrating the timecourse of partial hepatecomy *in vivo* in both WT and *Clspn^+/−^* mice. Seventy per cent of the liver was removed by surgical resection in 12-week-old male mice. 20 h or 36 h post-partial hepatecomy mice were humanely sacrificed and tissues collected. (**B**) *Clspn^+/−^* mice show increased liver regeneration after partial hepatecomy. Liver : body weight ratio at 20 h and 36 h post-partial hepatecomy in male WT (20 h *n* = 4, 36 h *n* = 4) and *Clspn^+/−^* (20 h *n* = 6, 36 h *n* = 7) mice. Analysed using an one-way ANOVA with Sidak *post-hoc* test. (**C**) *Clspn^+/−^* mice show increased levels of DNA damage after partial hepatecomy. Quantification of γH2AX positive pixels by IHC analysis in WT and *Clspn^+/−^* livers either 20 h or 36 h post-partial hepatecomy. Each dot represents one mouse and at least five fields of view were blind analysed per mouse. Representative images are shown. Analysed using an one-way ANOVA with Sidak *post-hoc* test. (**D**) Schematic diagram illustrating the acute DEN *in vivo* study in WT or *Clspn^+/−^* mice; 80 mg/kg DEN in 0.9% saline was administered IP to 8-week-old male mice. Twenty four hours or 48 h post-DEN mice were humanely sacrificed and tissues collected. (**E**) *Clspn^+/−^* mice show increased body weight loss after acute DEN treatment. Percentage change in body weight in WT (24 h *n* = 8, 48 h *n* = 8) and *Clspn^+/−^* (24 h *n* = 8, 48 h *n* = 8) mice 24 h or 48 h post-DEN injury. Analysed using an one-way ANOVA with Sidak *post-hoc* test. (**F**) *Clspn^+/−^* mice show increased liver damage after acute DEN treatment. Liver:body weight ratio at 24 h and 48 h post-DEN injury in male WT (24 h *n* = 8, 48 h *n* = 8) and *Clspn^+/−^* (24 h *n* = 8, 48 h *n* = 8) mice. Analysed using an one-way ANOVA with Sidak *post-hoc* test (*P < 0.05, **P < 0.01). (**G**) *Clspn^+/−^* mice show increased levels of DNA damage after acute DEN treatment. Quantification of γH2AX positive pixels by IHC analysis in WT and *Clspn^+/−^* livers either 24 h or 48 h post-DEN injury. Each dot represents one mouse and at least blinded five fields of view were analysed per mouse. Representative images are shown, with red dots in scatter plots indicating representative images chosen. *P < 0.05, **P  < 0.01 (one-way ANOVA with Sidak *post-hoc* test).

To first investigate the role of Claspin in the proliferative response to liver injury, we performed partial hepatectomies in *Clspn^+/−^* mice. The liver is known to rapidly regenerate over a period of 5 days in rodents following surgical resection of the liver [31]. Supporting a function for Claspin during liver regeneration, male *Clspn^+/−^* mice had a higher liver-to-body weight ratio relative to WT mice both 20 and 36 h following hepatectomy (Figure 4A,B). This is suggestive of a greater rate of hepatocyte proliferation following injury and was associated with enhanced levels of γH2AX staining in *Clspn^+/−^* mice (Figure 4C). We suggest that reduced Claspin levels lead to increased DNA replication stress and thereby also increase DNA damage. Given that proliferation is not restrained in *Clspn^+/−^* mice to the same extent as it is in WT, this could indicate that inhibition of cell cycle progression due to the DNA damage checkpoints is somewhat compromised in the *Clspn^+/−^* mice.

### *Clspn*^+/−^ mice are more susceptible to DEN-induced acute liver injury

The partial hepatectomy results demonstrate that Claspin is an important component of a regulatory mechanism controlling the hepatocyte proliferative response and the prevention of cellular and genomic damage. We, therefore decided to investigate whether Clspn haploinsufficiency could influence the development of liver cancer following N-nitrosodiethylamine (DEN) induced hepatocellular carcinoma (HCC) [32]. First we investigated whether Claspin regulates the acute hepatocyte response to DEN treatment (Figure 4D), a DNA alkylating agent causing DNA damage, hepatocyte death and subsequent compensatory proliferation. We hypothesised that *Clspn^+/−^* mice would be more susceptible to the acute liver injury induced by DEN. Indeed, acute DEN administration resulted in a significant decrease in the body weight of *Clspn^+/−^* mice compared with WT counterparts (Figure 4E), suggesting greater damage and parenchyma loss. Consistent with this, the liver-to-body weight ratio in *Clspn^+/−^* mice (Figure 4F) was also significantly reduced. This is suggestive of increased hepatocyte damage in the *Clspn^+/−^* mice following DEN treatment. Confirming this, we found that DEN-induced DNA damage (as measured by γH2AX staining) was significantly more pronounced in *Clspn^+/−^* mice (Figure 4G). Moreover, this damage persisted for a longer period of time in the *Clspn^+/−^* mice (Figure 4G), suggesting that DNA mutations remain unrepaired during the compensatory proliferation period, resulting in genomic instability and potentially malignant transformation.

### Increased susceptibility of hepatocellular carcinoma in *Clspn*^+/−^ mice

**Figure 5.**
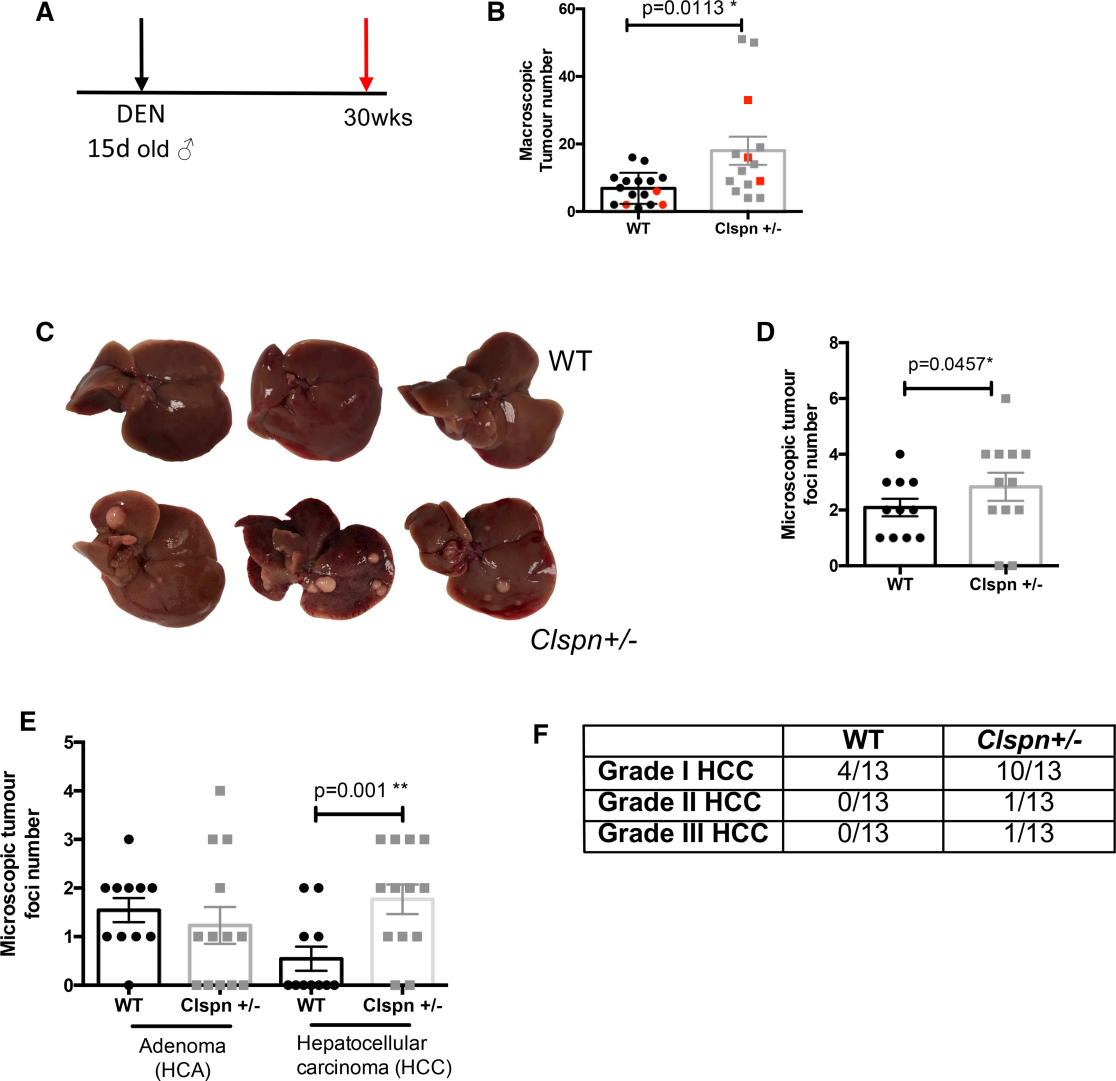
Increased susceptibility of DEN-induced hepatocellular carcinoma in *Clspn^+/−^* mice. (**A**) Schematic diagram illustrating the chronic DEN *in vivo* study in WT and *Clspn^+/−^* mice. Fifteen-day old mice were given 30 mg/kg DEN in 0.9% saline by IP injection. Black arrow indicates DEN administration and the red arrow indicates the time of study termination. (**B**,**C**) Livers from *Clspn^+/−^* mice show increased number of macroscopic tumours. Numbers of macroscopic tumours visible in WT (*n* = 16) and *Clspn^+/−^* (*n* = 14) livers 30 weeks after DEN administration were counted (**B**). Data represent mean ± SEM. *P < 0.05 (Unpaired Student's *t*-test). Representative images (**C**) of livers in WT and *Clspn^+/−^* livers 30 weeks after DEN administration. Red dots in (**B**) scatter plots indicate liver images used. (**D**) Numbers of microscopic tumour foci in WT (*n* = 11) and *Clspn^+/−^* (*n* = 12) livers 30 weeks after DEN administration were counted. Data represent mean ± SEM. *P < 0.05 (Unpaired Student's *t*-test). This is the number of foci counted in small histological section from three of the five lobes of the DEN-treated liver. (**E**) DEN-treated *Clspn^+/−^* mice have increased numbers of hepatocellular carcinoma. Histological quantification of tumours classified as either hepatocellular adenomas (HCA) or hepatocellular carcinomas (HCC) in liver sections from DEN-treated WT (*n* = 13) and *Clspn^+/−^* (*n* = 13) mice. An expert pathologist scored the pathology taken from a small histological section from three of the five lobes of the DEN-treated liver. Data represent mean ± SEM. *P < 0.05, **P < 0.01 (Unpaired Student's *t*-test). (**F**) Table summarising the grade of HCC observed in the DEN-treated WT (*n* = 13) and *Clspn^+/−^* (*n* = 13) mice.

We next investigated whether reduced Claspin levels affect tumorigenesis in the chronic model of DEN-induced HCC. DEN was administered to 15-day-old WT and *Clspn^+/−^* mice. Mice were then euthanised 30 weeks post-DEN administration (Figure 5A), and macroscopic liver tumours were counted (Figure 5B). Importantly, there was a significantly higher number of both macroscopic and microscopic liver tumours in *Clspn^+/−^* livers compared with WT littermates (Figure 5B–D). This was also coupled with a higher liver-to-bodyweight ratio in the *Clspn^+/−^* animals (Supplementary Figure S3D), suggesting loss of Clapsin was at least, in part, responsible for the earlier onset of tumours in this model.

Detailed histological analysis of liver tissue sections revealed higher numbers of mitotic bodies in tumours from *Clspn^+/−^* mice (Supplementary Figure S3E). Moreover, while the numbers of adenomas were broadly equivalent between the two mice strains (17 foci from 13 WT mice, 16 foci from 13 *Clspn^+/−^* mice in total from the liver sections analysed), the more malignant HCC numbers were dramatically different. Here, the 13 *Clspn^+/−^* mice had 23 HCCs in total from the liver sections analysed, including one mouse with 2 grade II and one mouse with 1 grade III HCC, versus only six grade I HCC in the WT mice (Figure 5E,F and Supplementary Figure S3F–H).

Together these data suggest that either a loss of Claspin could be responsible for an earlier onset of tumourigenesis in the DEN model and/or, the loss could increase the probability that cells undergo malignant transformation, increasing adenoma–carcinoma progression.

## Discussion

Here, we show for the first time that Claspin haploinsufficiency has profound effects on multiple biological systems. Reduced Claspin expression results in the earlier onset of both haematopoietic and solid tumours, a predisposition to NASH and, reduced female fertility.

A lack of Claspin likely alters two fundamental, intrinsically linked pathways within the cell: the DNA damage response and cell proliferation. It is reasonable to suggest that the phenotypes we observe are a result of the perturbation of both pathways, and that outcomes will likely be different in different cell types. For example, hepatocytes are highly proliferative, therefore, in a confounding environment of Claspin insufficiency, damage either by DEN or an MCD diet could drive hyperproliferation resulting in the liver phenotypes we describe. Moreover, in an aging liver, hyperproliferation coupled with ongoing endogenous DNA damage may drive the NASH observed in *Clspn^+/−^* mice. In contrast, lymphoid cell turnover is slow and in a low Claspin environment with underlying genomic instability, the damage accumulating in an aged animal could result in hyperplasia. The most extreme example of slow proliferation is the oocyte, a cell that has remained at the same stage of its cell cycle since before birth. In this situation, it is plausible that by impairing a DNA damage repair pathway, the cumulative damage caused over months (or years), is insurmountable resulting in either a permanent cell cycle block or embryonic deformities. Moreover, the lack of proliferation in oocytes prevents the dilution of epigenetic markers, thereby promoting the inheritance of defects. We suggest that Claspin could fulfil a unique role within the cell, acting to directly couple DNA damage responses to proliferation, explaining why the consequences of reduced Claspin expression is context and cell-type dependent.

Focusing on tumorigenesis, in this report, our data reflect the consequences of a loss of Claspin (and likely downstream effects on CHK1 activity and DNA replication) at the early stages of tumorigenesis. Our use of germline mouse models, mean defects in checkpoint kinase signalling will be present when tumours first start to develop. We, therefore, hypothesise that low levels of Claspin result in a failure to mount an efficient DNA replication stress response. This would lead to increased genomic instability and could be the cause of the earlier onset of cancer we have observed in the Eμ-Myc lymphoma model [12] and here in the DEN model of HCC. Furthermore, given that we also see an increased susceptibility to steatosis using the MCD diet model and in aging *Clspn^+/−^* mice (Figure 3), we cannot rule out that a combination of hepatocyte proliferation and fat droplets may co-operate to accelerate tumour development. Please note, we were unable to test this using a Eμ-Myc/*Clspn^+/−^* mouse, since the numbers of animals required to generate sufficient mice of this genotype for a meaningful study would be unethical. Mutations in Claspin have been described in familial and sporadic breast and ovarian cancers [33]. One mutant (I783S) found in both cell lines and tumour samples [34] was found to result in loss of function, hence it is hypothesised that CLSPN mutation or loss may result in a predisposition to cancer.

In contrast with the early stages of tumorigenesis, later stage cancer cells need to acquire mechanisms that allow them to cope with high levels of genomic instability, and consequently can become addicted to the ATR/CHK1 pathway [2,5]. Though we did not test this directly, this addiction could explain the variable correlation between Claspin expression and patient survival in different tumour types (Figure 1B–D; Since these patients’ data reflect the situation in more advanced tumours, it does not necessarily reveal events earlier in the process of tumorigenesis). We propose that where high Claspin expression is associated with poor patient survival, this reflects its role in allowing these tumours to deal with high levels of DNA replication stress. In contrast, where low levels of Claspin are associated with poor survival this may indicate either intrinsically lower levels of genomic instability or that the tumours have engaged other pathways to deal with rapid unstable proliferation.

Focusing on female meiosis, CHK1/CHK2 have both been implicated in the removal of damaged oocytes from the ovary [35,36], and independent of this, acute depletion of CHK1 has been shown to arrest porcine oocytes in MI [37]. However, a functional role for Claspin in oocyte meiosis was previously unexplored. Here, we show that the reproductive potential of *Clspn^+/−^* females is significantly reduced (Figure 2). Furthermore, and most remarkably, we find that this defect is transmitted from mother to daughter. Given that all oocytes are generated *in utero* [20], we suggest that this maternal influence is either due to a failure to sufficiently repair *in utero* DNA damage in primordial germ cells and/or, to inherited epigenetic memory [21]. It is, therefore, conceivable that the impaired DNA damage response in *Clspn^+/−^* females is analogous to DNA damage caused by alcohol consumption, obesity and smoking while primordial germ cells are being laid down in the foetal ovary, an effect which could be confounded by damage to the mitochondrial genome, and therefore only passed on by the mother [38]. Claspin is required for the ATR-dependent phosphorylation of Brca-1 [39]. It is Interesting, therefore, that *Brca1-*deficient mice are sub-fertile due to unresolved double-stranded DNA breaks in their oocytes and, women with germline mutations in *BRCA1* have depleted ovarian reserves [40].

Taken together our data demonstrate for the first time that Claspin can function as a tumour suppressor and prevent both cancer development and fatty liver disease. Moreover, Claspin plays a critical role in the production of an oocyte capable of completing meiosis to produce an egg capable of producing young. It is essential that we obtain a better understanding of Claspin regulation, as this could not only lead to the development of Claspin as a patient stratification biomarker where ATR and CHK1 inhibitors are used in the clinic, but could have important implications for the use of such compounds in women of child-bearing age.

## Methods

### Ethics statement

All mouse experiments were approved by Newcastle University's Animal Welfare and Ethical Review Board. All procedures, including the of breeding genetically modified mice were carried out under project and personal licenses approved by the Secretary of State for the Home Office, under the United Kingdom's 1986 Animal (Scientific Procedures). Animals were bred in the Comparative Biology Centre, Newcastle University animal unit, according to the FELASA Guidelines.

### Mouse models

*Clspn^+/−^* mice were generated by the Knockout Mouse Project using C57Bl/6 ES cells, and obtained via RIKEN (Saitama, Japan). *Clspn^+/−^* mice were maintained on a pure C57Bl/6n background. In all experiments, the relevant pure C57Bl/6 (WT) strain was used as a control. In all experiments, except those involving oocytes, mice were designated to an experimental group dependent on their genotype. For all murine oocyte work, experimental work was performed following the blinding of groups/genotypes. Unless otherwise stated, procedures using animal models did not require any use of anaesthesia.

### Oocyte methods

The ovaries of sexually mature 6-week-old mice were collected into warmed M2 media (Sigma) for oocyte collection and oocyte grading. Importantly, all collection, handling and culture methods were carried out by the same experienced operator, who was blind to the genotype of the mouse. For oocyte collection, the follicles of each ovary were punctured with a sterile needle to release their contents into fresh M2 media supplemented with 30 nM 3-isobutyl-1-methylxanthine (IBMX; Sigma; to maintain prophase I arrest for initial assessment). Oocytes were then stripped of their cumulus cells mechanically using a pipette, and transferred to clean M2 + IBMX to be scored for known indicators of oocyte competency. Spherical oocytes, of a mature size, with a central Germinal Vesicle and uniform granulation were considered ‘healthy’ and at the correct stage of their cell cycle for meiosis I resumption and subsequent ovulation. In contrast, oocytes that fell out of these criteria and were considered to be either ‘immature’, unhealthy, or to have failed to arrest their cell cycle at the correct time point ‘failed prophase I arrest’. Oocytes that failed to arrest in prophase I were counted and discarded. All other oocytes were separated into pools of ‘healthy’, ‘immature’ and ‘unhealthy’ oocytes. Pools were then washed into fresh M2 media, now in the absence of IBMX, triggering spontaneous cell cycle resumption. Within each pool, oocytes were subsequently scored for Germinal Vesicle Breakdown (an indicator of successful cell cycle resumption, ∼1–1.5 h post washing) and following this, Polar Body extrusion (an indicator of completion of meiosis 1, ∼9–12 h post washing). All handling and culture were carried out at 37°C. On each experimental day, the ovaries of both WT and *Clspn^+/−^* mice were assessed alongside each other.

### Embryo isolation and visualisation using computerised tomography (CT) scanning

WT or *Clspn^+/−^* pairings were mated overnight. Then, male mice were removed (E0.5) and the females were checked for a mucus vaginal plug. At E13.5, female mice were humanely sacrificed by cervical dislocation and the embryos harvested. The yolk sac was removed for genotyping and the embryos fixed in 10% Formalin solution (HT501850, Sigma) for 24 h. Following this, the embryos were embedded in 1% (w/v) agarose and stained with Lugol's solution (1.09261, Sigma) for a further 24 h. Following this, micro-CT was performed on the embryos in collaboration with the Newcastle Preclinical *In Vivo* Imaging Facility using the Skyscan 1176, Bruker-microCT system. All samples were acquired at an isotropic resolution of 9 μm using the following parameters: a source voltage of 45 kV, source current of 550 μA, 1315 ms exposure time, 0.5 m  Aluminium filter, 0.3° rotation step using 360 projections, 4 frame averaging. The total scan duration was 3 h. Image reconstruction was performed using NRecon software (Bruker).

### *In vivo* models of liver injury and hepatocellular cancer

80 mg/kg DEN (N0258, Sigma) in 0.9% saline was administered intraperitoneally (IP) to 8-week-old male mice for the acute liver injury studies. In this case, mice were humanely sacrificed by cervical dislocation 24 h or 48 h post-DEN and tissues collected. To induce liver cancer, day 15 mice were given 30 mg/kg DEN in 0.9% saline by IP injection. Mice were humanely sacrificed by cervical dislocation, and liver tissue was harvested at 30 weeks post-DEN.

### Partial hepatectomy

Seventy per cent liver partial hepatectomy was performed on 10 to 12-week-old male littermates under isoflurane anaesthesia as previously described [41]. Appropriate buprenorphine pain relief was given. Twenty hours and 36 h post-surgery the mice were humanely sacrificed by cervical dislocation and liver tissue was harvested and used for downstream analysis.

### Methionine and choline-deficient dietary modification

Standard chow was replaced in 12-week-old male mice with methionine and choline-deficient (MCD) diet (A02082002BR Research Diets, U.S.A.) or the matched control diet (A02082003BY, Research Diets, U.S.A.). Dietary modification persisted for either 2 or 4 weeks, at which point mice were humanely sacrificed by cervical dislocation and liver tissue was harvested and used for downstream analysis.

### Antibodies

Antibodies used were β-Actin (A5441 Sigma), γH2AX (9718 Cell Signaling) and CD45R (ab18197). Antibodies to the murine form of Claspin were generated by Moravian Biotechnologies. Anti-rabbit IgG (A6154 Sigma and 7074 Cell Signaling) and anti-mouse IgG (A9044 Sigma) HRP-linked secondary antibodies were used for western blot detection.

### Western blotting

Whole-cell extracts were prepared from WT or *Clspn^+/−^* ear fibroblasts or extracted directly from snap frozen pieces of tumour or liver tissue. Snap frozen tumour or tissue was lysed in PhosphoSafe™ Extraction Reagent using the Precellys24 ceramic mix bead tubes (431-0170, Stretton Scientific Ltd) in a Precellys®24 homogeniser (Stretton Scientific Ltd) at 6500 rpm for 30 s, and then extracted according to the PhosphoSafe™ Extraction Reagent (71296, Merck Millipore) manufacturer's instructions. In the case of tumour cell suspensions, cell pellets were washed with ice-cold PBS, and lysed using PhosphoSafe™ Extraction Reagent (Merck-Millipore, Watford, U.K.), according to the manufacturer's protocols. Protein quantification was undertaken using the BCA protein assay, and samples resolved by standard denaturing SDS–PAGE gels. Samples were transferred onto PVDF membranes (GVWP04700, Merck-Millipore) before being probed with the primary antibody. Horseradish peroxidase-conjugated secondary antibodies (anti-mouse; Sigma, U.K., anti-rabbit; Sigma, U.K.) and enhanced chemiluminscence reagent (32106 Thermo-scientific, U.K.) were used for detection.

### Immunohistochemistry

Formalin-fixed tumour or liver tissues were paraffin-embedded and serial sections were cut by the Molecular Pathology Node, Cellular Pathology, Royal Victoria Infirmary, Newcastle-Upon-Tyne. H&E staining was also undertaken by the Molecular Pathology Node.

Formalin-fixed paraffin-embedded tumour and liver sections were dewaxed and hydrated. Endogenous peroxidase activity was blocked with hydrogen peroxide and antigen retrieval was achieved using 1 mM EDTA. Tissue was blocked using an Avidin/Biotin Blocking Kit (SP-2001, Vector Laboratories, Peterborough, U.K.) followed by 20% swine serum in PBS and then incubated with primary antibodies overnight at 4 °C. The following day, slides were washed and incubated with biotinylated swine anti-rabbit (E0353, Dako, U.K.) followed by Vectastain Elite ABC Reagent (PK7100, Vector Laboratories). Antigens were visualised using DAB peroxidase substrate kit (SK4001, Vector Laboratories) and counterstained with Mayer's haematoxylin. Immuno-stained cells were imaged using a DFC310 FX microscope (Leica Microsystems) and the images blinded (coded) prior to analysis by an independent party. At least five images per tissue at ×100 magnification (10× lens and 10× eye piece) were analysed using brown/blue pixel intensity using Adobe Photoshop.

### Kaplan–Meier analysis of patient survival times

Analysis of whether CLSPN mRNA expression levels correlate with human cancer patient survival was performed using the pan-cancer RNA Seq database at kmplot.com [15]. With CLSPN as the gene search terms, the following settings were used: Split patients by: Auto select best cutoff; ‘Compute median survival’ and ‘Censore at Threshold’ both selected; Follow up threshold set to ‘All’; Survival set to: OS (*n* = 7462); Analysis was not restricted to different subtypes (Set to ‘All’); Analysis was not restricted based on cellular content (Set to ‘All’). Data presented is for cancer types where the log rank *P-*value was <0.05. Please see Supplementary Data File S1 for the full analysis of all cancer types.

### Statistical analysis

GraphPad Prism software (http://www.graphpad.com, V6.0) was used for statistical analysis. In all figures, the data represented is the mean ± SEM. The statistical test (and any *post-hoc* test) used is stated in each figure legend. We have used parametric approaches such as Unpaired Student's *t*-tests, one-way ANOVA and Mantel–Cox tests to calculate *P-*values, and all statistical assumptions were fulfilled for each of the tests used. (*P-*values of *P* < 0.05 were considered signiﬁcant).

## Data Availability

The authors are happy to provide all original data, and for this to be shared on Figshare as appropriate.

## Open Access

Open access for this article was enabled by the participation of University of Liverpool in an all-inclusive *Read & Publish* agreement with Portland Press and the Biochemical Society under a transformative agreement with JISC.

## Abbreviations

ATR: ataxia telangiectasia and Rad3 related
CHK1: checkpoint kinase 1
CT: computerised tomography
HCC: hepatocellular carcinoma
IP: intraperitoneally
MCD: methionine and choline deficient
MI: meiosis I
MII: meiosis II
NAFLD: non-alcoholic fatty liver disease
PB1: 1st polar body
VLDL: very-low-density lipoprotein
WT: wild-type
