# Supplementary material for: Claspin haploinsufficiency leads to defects in fertility, hyperplasia and an increased oncogenic potential
Source: Biochem J. 2022 Oct 14;479(19):2115–30. doi: 10.1042/BCJ20220101 (PMC9704638; doi:10.1042/BCJ20220101)
Supplement: Supplementary Material [file BCJ-479-2115-s1.pdf]

Supplementary information:

Claspin haploinsufficiency leads to defects in fertility, hyperplasia and an increased oncogenic potential

Suzanne Madgwick[1], Saimir Luli[2], Helene Sellier[1], Jacqueline A. Butterworth[1], Jack Leslie[3], Adam J. Moore[1], Emma K. Corbin[1], Adrian I. Yemm[1], Robson T. Chiremba[1], Dina Tiniakos[3], Fiona Oakley[3], Neil D. Perkins[1*] and Jill E. Hunter[1*]

[1]Newcastle University Biosciences Institute

Wolfson Childhood Cancer Research Centre

Level 6, Herschel Building

Newcastle University

Brewery lane

Newcastle upon Tyne, NE1 7RU, UK

[2] Preclinical *In vivo* Imaging Facility,

[3]Newcastle Fibrosis Research Group, Newcastle University Biosciences Institute

Faculty of Medical Sciences

Newcastle University

Newcastle Upon Tyne, NE2 4HH, UK

* joint corresponding authors

Tel. 0191 2082245 Email: neil.perkins@ncl.ac.uk

Email: jill.hunter@ncl.ac.uk

Keywords: Claspin, hyperplasia, oocyte, genomic instability, replication stress

Figure S1 (related to Figure 2):

(A) Scatter plot showing the number of viable pups born in the first 100 days of mating in a range of different matings: WT male and WT females (n=3), *Clspn*$^{+/-}$ male and *Clspn*$^{+/-}$ females (n=8) , WT male and *Clspn*$^{+/-}$ females (n=4) or *Clspn*$^{+/-}$ male and WT females (n=4). Analysed using an ONE-way Anova with Sidak post-hoc test.

(B & C) Scatter plots showing the weight of the left (B) and right (C) ovary in WT and *Clspn*$^{+/-}$ mice and indicating the maternal genotype. WT females from WT mother (n=18), *Clspn*$^{+/-}$ females from WT mother (n=13), WT females from *Clspn*$^{+/-}$ mother (n=18), *Clspn*$^{+/-}$ females from *Clspn*$^{+/-}$ mother (n=18). Analysed using an ONE-way Anova with Sidak post-hoc test, no differences were detected.

(D) Scatter plot showing an increase in immature oocytes in 6 week old *Clspn*$^{+/-}$ females. WT (n=9), *Clspn*$^{+/-}$ (n=11). Unpaired Student's t-test were performed but no significant differences were detected.

(E) Scatter plot showing the number of unhealthy oocytes in 6 week old WT and *Clspn*$^{+/-}$ females. WT (n=9), *Clspn*$^{+/-}$ (n=11). Unpaired Student's t-test were performed but no significant differences were detected.

(F) Scatter plot showing an increase in oocytes that failed to arrest in Prophase I in 6 week old *Clspn*$^{+/-}$ females. WT (n=9), *Clspn*$^{+/-}$ (n=11). Unpaired Student's t-test were performed but no significant differences were detected.

(G) Examples of oocytes that are i, 'immature': a slightly smaller oocyte with an uneven appearance to the membrane, uneven granulation patterning to the cytoplasm and zona pellucida, and a germinal vesicle that lacks a smoothed spherical appearance. Oocytes such as this are highly unlikely to resume their cell cycle. ii, 'unhealthy': oocyte with swollen appearance and an off centre germinal vesicle, the zona pellucida appears thinned over some areas. During handling, oocytes such as this lack the rigidity of a 'healthy' oocyte and readily become misshapen, they often do resume their cell cycle, but then fail to extrude a PB1 and

complete meiosis I. iii + vi, ‘healthy’ full size oocytes, with uniform cytoplasmic granulation, smoothed inner membrane (the oocytes true membrane), clear smoothed zona pellucida (outer ring), and central spherical germinal vesicle.

(H) Images of E13.5 embryos from WT or *Clspn*$^{+/-}$ matings, taken at the time of harvest. Four embryos on the left hand side are harvested from WT males mated with WT females are of the expected size and embryonic development. Three embryos on the right hand side are from *Clspn*$^{+/-}$ males mated with *Clspn*$^{+/-}$ females and show two embryos that are underdeveloped, potentially with limb deformities, and a final embryo which appears to have arrested at a very early stage of development.

Figure S2 (related to Figure 3):

(A) 18 month old *Clspn*$^{+/-}$ mice spontaneously develop B-cell lymphoma. Table summarizing the tumour incidence in various organs in aged WT (n=5) and *Clspn*$^{+/-}$ (n=9) mice.
(B-C) Weight of the mesenteric lymph node from 6 month old WT (n=7) and *Clspn*$^{+/-}$ (n=8) (B) and 12 month old (C) WT (n=10) and *Clspn*$^{+/-}$ (n=12) mice. Unpaired Student’s t-test were performed but no significant differences were detected.

(D-I) Weight of the indicated lymph nodes from 12 month old WT (n=10) and *Clspn*$^{+/-}$ (n=12) (D-F) and 18 month old (G-I) WT (n=5) and *Clspn*$^{+/-}$ (n=9) mice. Unpaired Student’s t-test were performed but no significant differences were detected.

Figure S3 (related to Figures 3 and 5):

(A and B) The increase in bodyweight over the lifespan of WT (n=7) and *Clspn*$^{+/-}$ (n=8) males (A) and WT (n=6) and *Clspn*$^{+/-}$ (n=7) females (B).
(C) 12 month old *Clspn*$^{+/-}$ mice develop macro- and micro- steatohepatitis. Scatter plots indicating an increased liver weight and liver:bodyweight ratio in aged WT (n=4) *Clspn*$^{+/-}$ (n=5) mice. Analysed using an Unpaired Student’s t-test
(D) Liver:body weight ratio at 30 wks post DEN treatment in male WT (n=16) and *Clspn*$^{+/-}$ (n-

14) mice. Analysed using Unpaired Student's t-test.

(E) Summary table of the pathological analysis of liver tumours. This includes the presence of mitotic bodies, nuclear hyperplasia, irregular nuclei and proteoglycan globules as well the nuclear: cytoplasmic ratio.

(F) Representative liver IHC images from 30 wks DEN treated WT animals. The images show the presence of adenomas (HCA) but not hepatocellular carcinomas in these animals.

(G) Representative liver IHC images from 30 wks DEN treated *Clspn*$^{+/-}$ animals. The images show the presence of hepatocellular carcinomas (HCC) in these animals, including a Grade II HCC. Red arrows denote the presence of proteoglycans and black arrows denote the presence of mitotic bodies.

(H) Examples of adenomas (HCA), Grade I hepatocellular carcinomas (Gr1 HCCs) and Grade II hepatocellular carcinomas Gr2 HCCs.

Supplementary Data File 1

Survival analysis for all cancer types for Clspn expression, using the pan-cancer RNA Seq database at kmplot.com

Madgwick et al., Figure S1

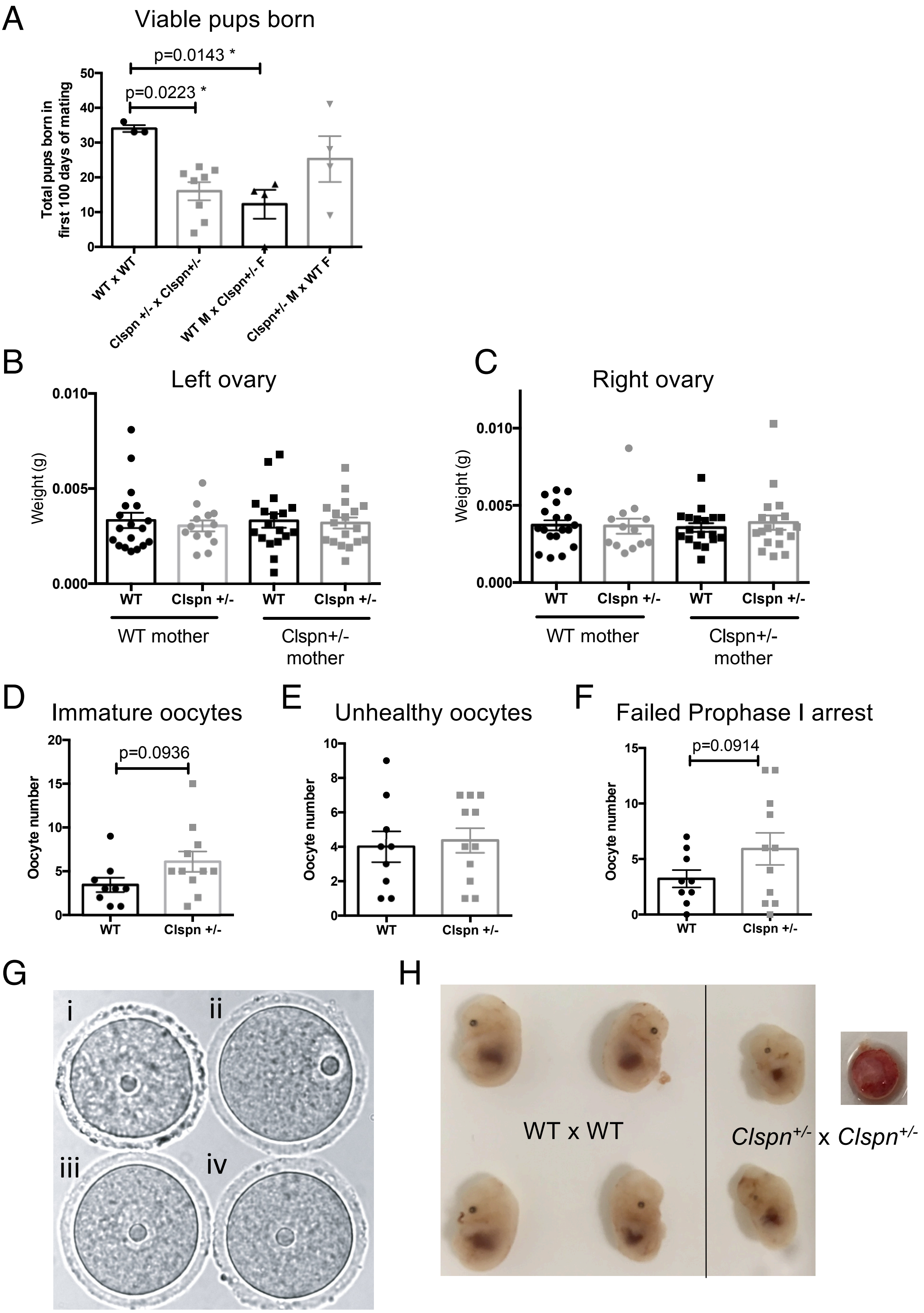

Madgwick et al., Figure S2 (Related to Figure 3)

A

SITE	HYPERPLASIA													
	WT					Clspn+/-								
	1	2	3	4	5	1	2	3	4	5	6	7	8	9
Mesenteric LN						x	x			x	x	x		
Sml intestine/colon						x	x							
Inguinal LN							x							
Brachial LN							x							
Cervical LN							x				x			
Spleen							x							
Thymus							x				x			
Pancreas							x							
No hyperplasia	x	x	x	x	x			x	x				x	x

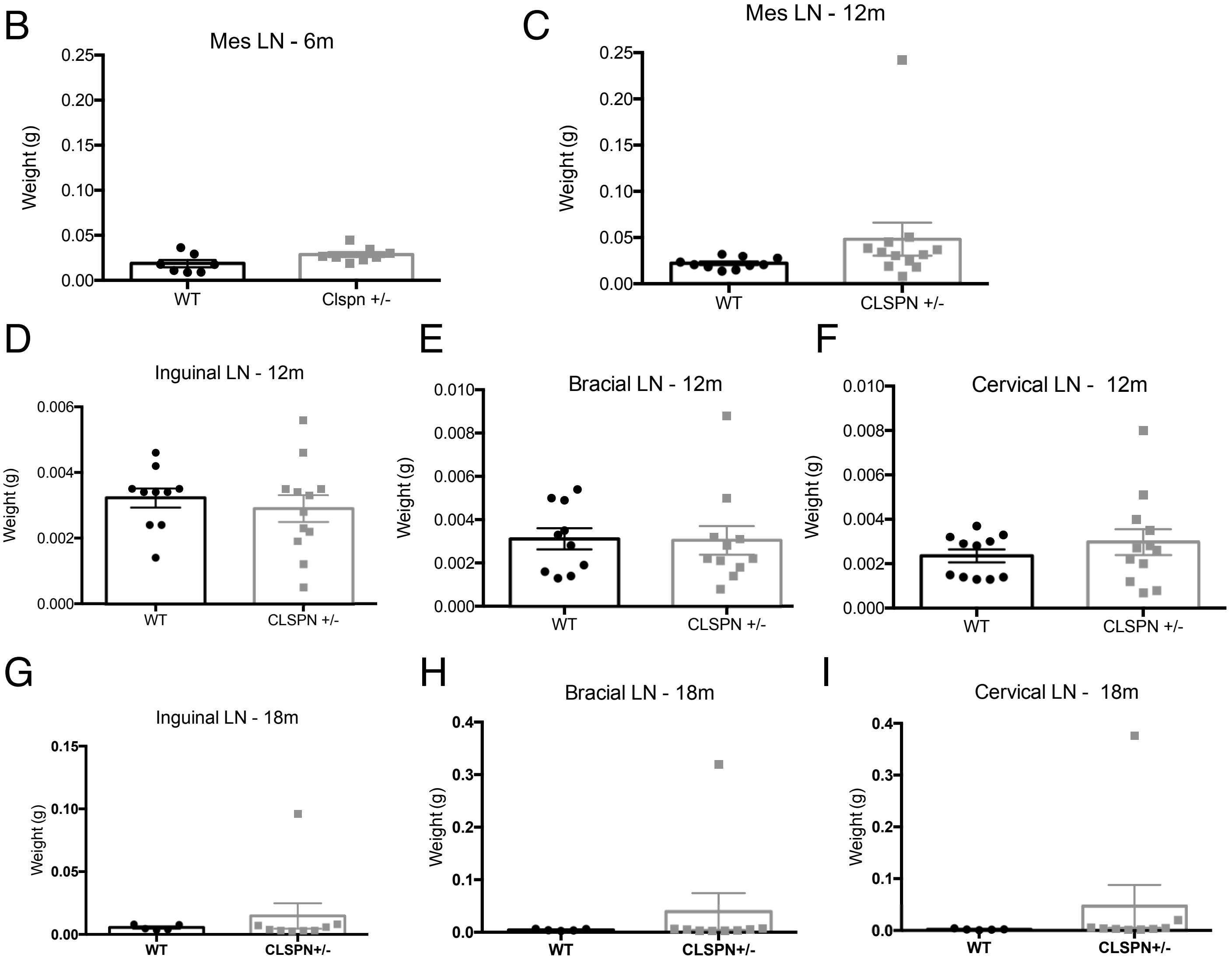

Madgwick et al., Figure S3

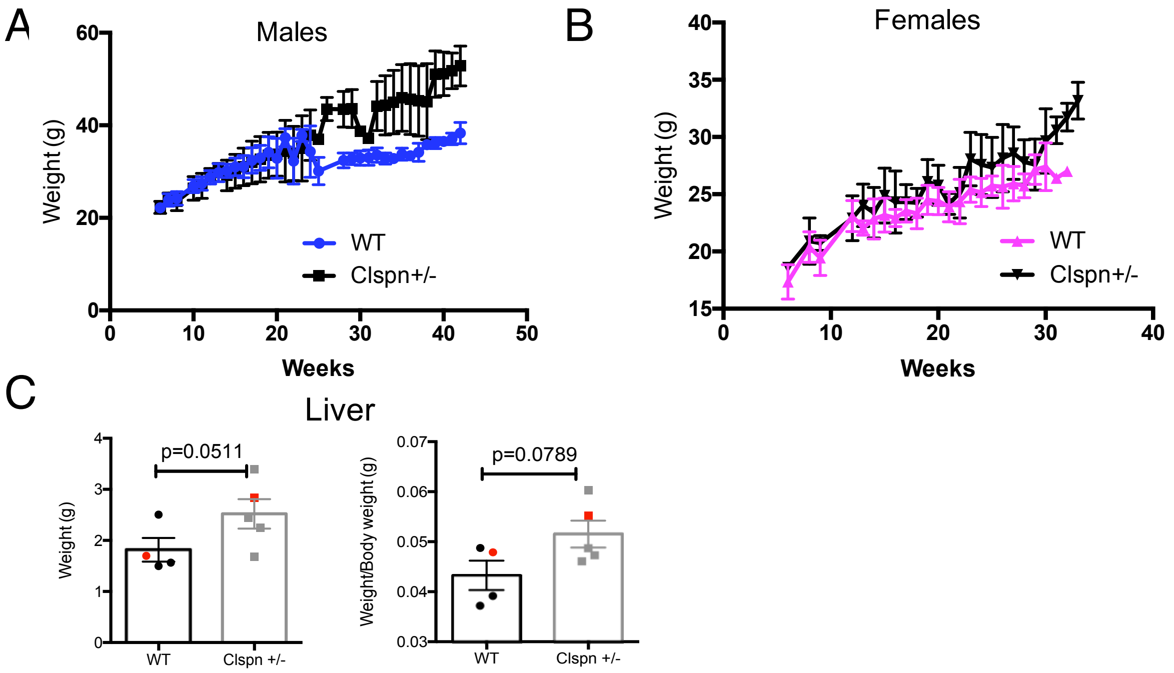

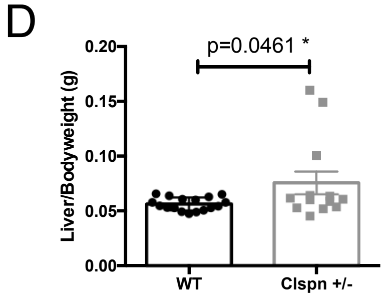

E

	WT	*Clspn+/-*
**1-2 mitotic bodies**<br>**3+ mitotic bodies**<br>**Total showing mitotic bodies**	6/22 (27%)<br>0/22 (0%)<br>**6/22 (27%)**	15/34 (44%)<br>2/34 (6%)<br>**17/35 (50%)**
**Increased nuclear/cytoplasmic ratio**	22/22 (100%)	34/34 (100%)
**Nuclear hyperplasia**	15/22 (68%)	26/34 (76%)
**Irregular nuclei**	10/22 (45%)	8/34 (24%)
**Proteoglycan globules**	20/22 (90%)	34/34 (100%)

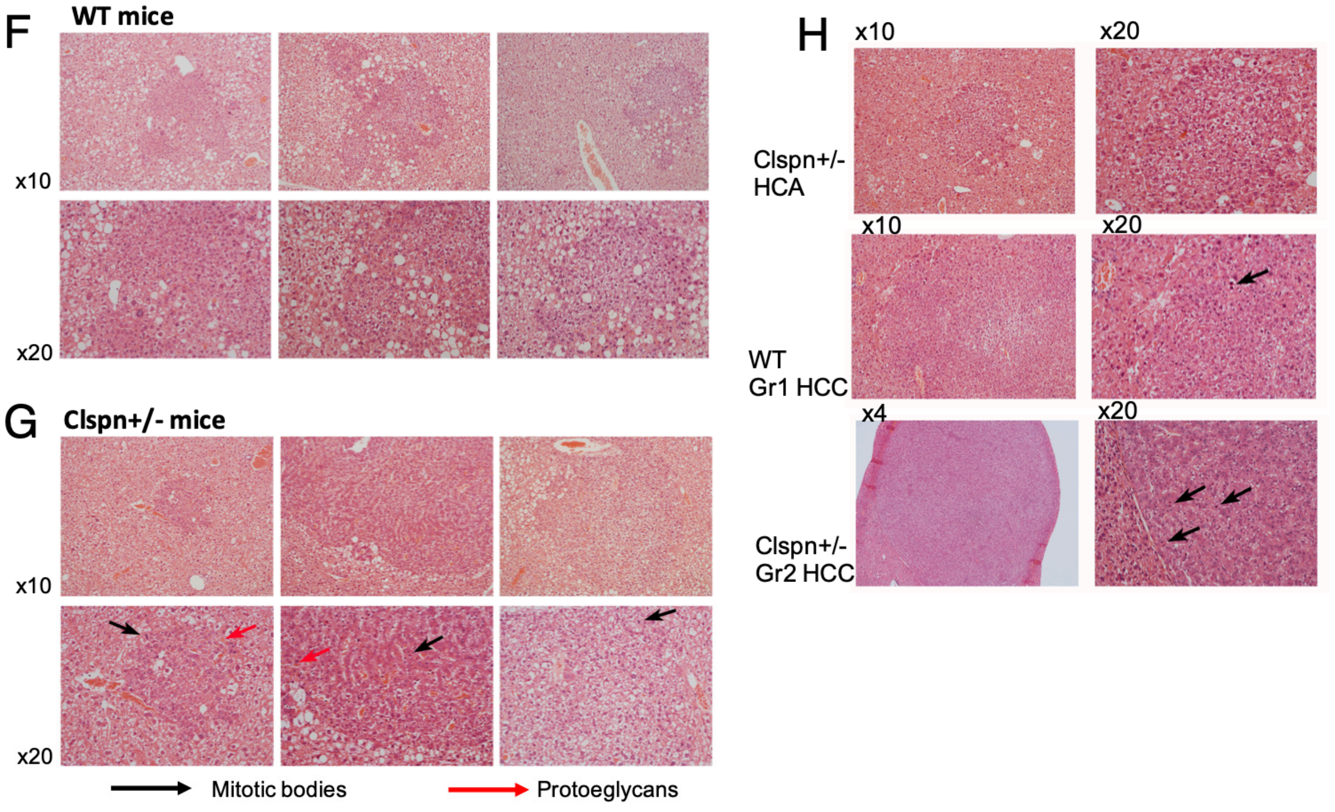

Kaplan-Meier Plotter

Pan-cancer RNA-seq

Pan-cancer

KM plotter | Home | Upload | Download | Updates | Contact

The desired RNAseq ID is valid: CLSPN (-),

**RNAseq ID:**	CLSPN -
**Survival:**	OS
**Auto select best cutoff:**	checked
**Follow up threshold:**	all
**Censore at threshold:**	checked
**Compute median over entire database:**	false
**Cutoff value used in analysis:**	92
**Expression range of the probe:**	8 - 2060
**Invert HR values below 1:**	not checked

Restrictions

Tumor type: Bladder Carcinoma

Restrict analysis to subtypes...

Stage:	all
Gender:	all
Race:	all
Grade:	all
Mutation burden:	all
Neoantigen load:	all

Restrict analysis based on cellular content...

Basophils:	all
B-cells:	all
CD4+ memory T-cells:	all
CD8+ T-cells:	all
Eosinophils:	all
Macrophages:	all
Mesenchymal stem cells:	all
Natural killer T-cells:	all
Regulatory T-cells:	all
Type 1 T-helper cells:	all
Type 2 T-helper cells:	all

Results

P value: 0.177
FDR: 100%

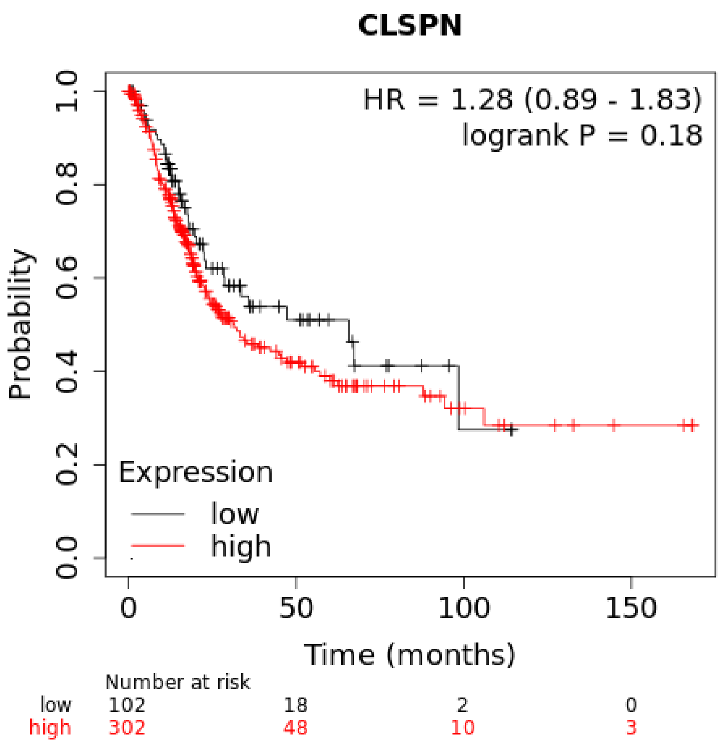

Download plot as a PDF

Auto cutoff plot

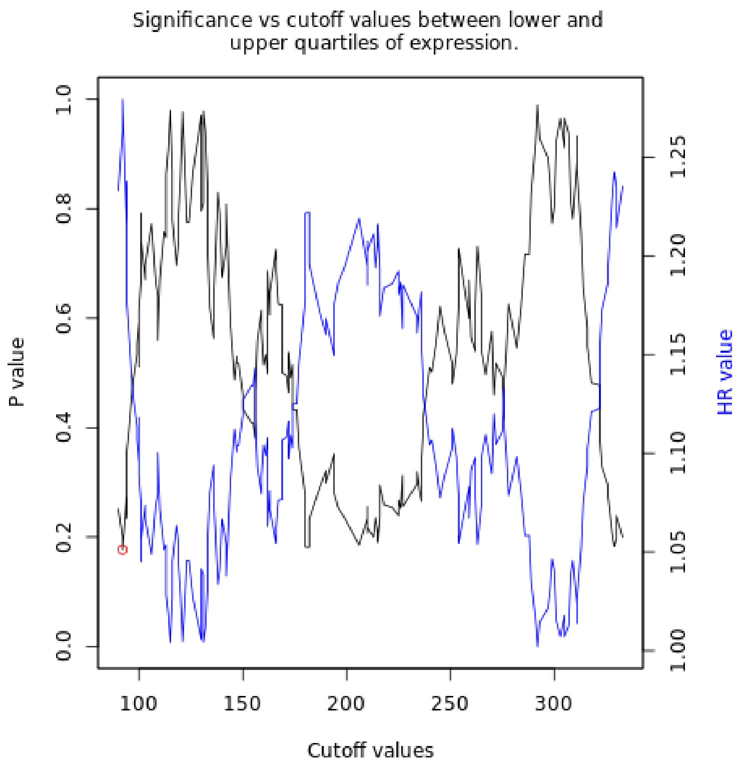

Download p values vs. cutoff table

Median survival

Low expression cohort (months)	High expression cohort (months)
65.7	31.63

Click here for a permanent link

RNAseq ID: CLSPN -
Survival: OS

**Auto select best cutoff:**	checked
**Follow up threshold:**	all
**Censore at threshold:**	checked
**Compute median over entire database:**	false
**Cutoff value used in analysis:**	82
**Expression range of the probe:**	2 - 2227
**Invert HR values below 1:**	not checked

Restrictions

Tumor type: Breast cancer

Restrict analysis to subtypes...

Stage:	all
Gender:	all
Race:	all
Grade:	all
Mutation burden:	all
Neoantigen load:	all

Restrict analysis based on cellular content...

Basophils:	all
B-cells:	all
CD4+ memory T-cells:	all
CD8+ T-cells:	all
Eosinophils:	all
Macrophages:	all
Mesenchymal stem cells:	all
Natural killer T-cells:	all
Regulatory T-cells:	all
Type 1 T-helper cells:	all
Type 2 T-helper cells:	all

Results

P value: 0.0935
FDR: 100%

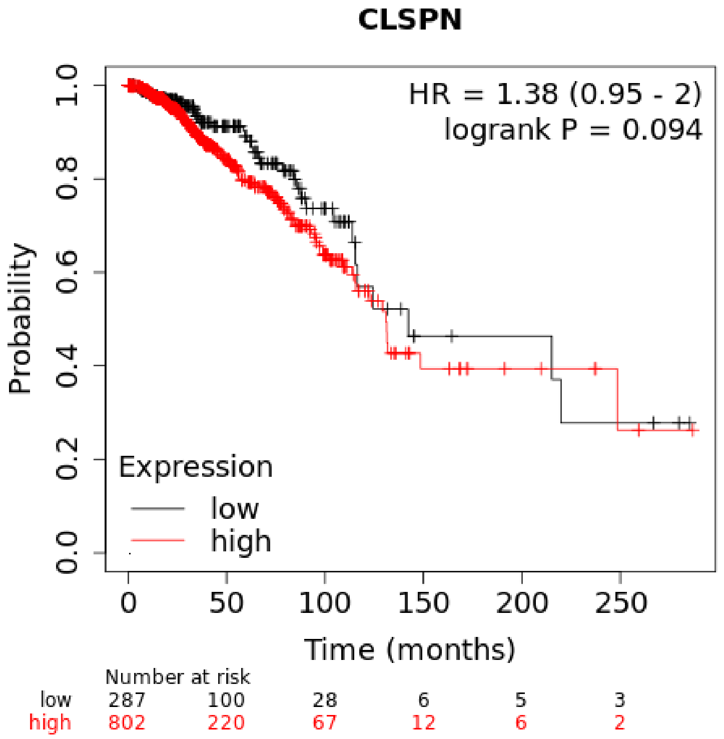

Download plot as a PDF

Auto cutoff plot

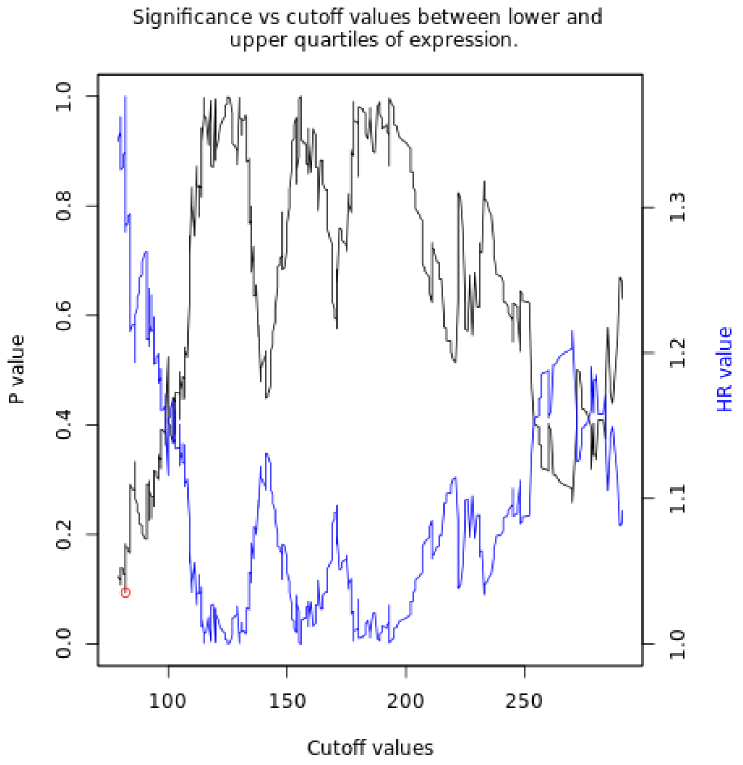

Download p values vs. cutoff table

Median survival

Low expression cohort (months)	High expression cohort (months)
142.23	130.87

Click here for a permanent link

RNAseq ID: CLSPN -
Survival: OS
Auto select best cutoff: checked
Follow up threshold: all
Censore at threshold: checked
Compute median over entire database: false
Cutoff value used in analysis: 407
Expression range of the probe: 23 - 1501
Invert HR values below 1: not checked

Restrictions

Tumor type: Cervical squamous cell carcinoma

Restrict analysis to subtypes...

Stage: all
Gender: all
Race: all
Grade: all
Mutation burden: all
Neoantigen load: all

Restrict analysis based on cellular content...

Basophils: all
B-cells: all
CD4+ memory T-cells: all
CD8+ T-cells: all

Eosinophils:	all
Macrophages:	all
Mesenchymal stem cells:	all
Natural killer T-cells:	all
Regulatory T-cells:	all
Type 1 T-helper cells:	all
Type 2 T-helper cells:	all

Results

P value: 0.1004
FDR: 100%

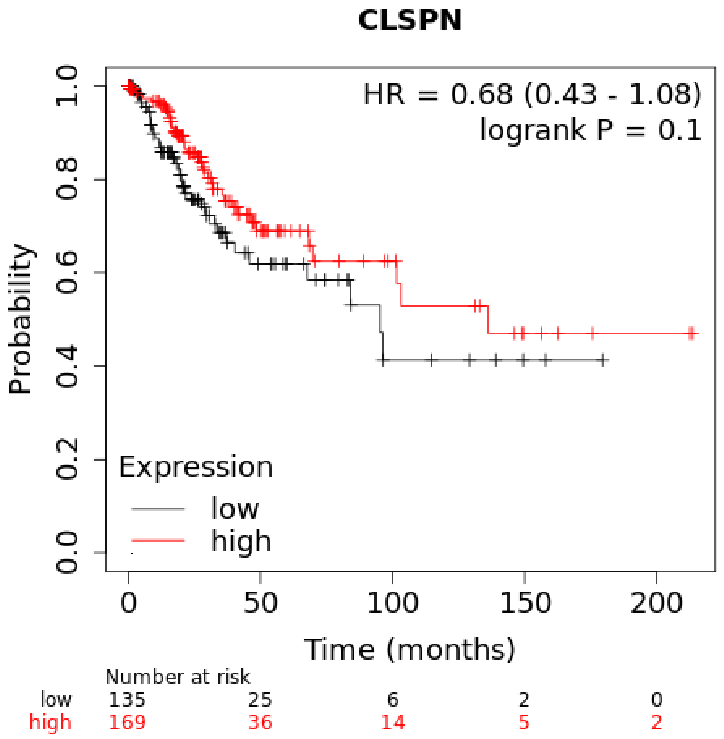

Download plot as a PDF

Auto cutoff plot

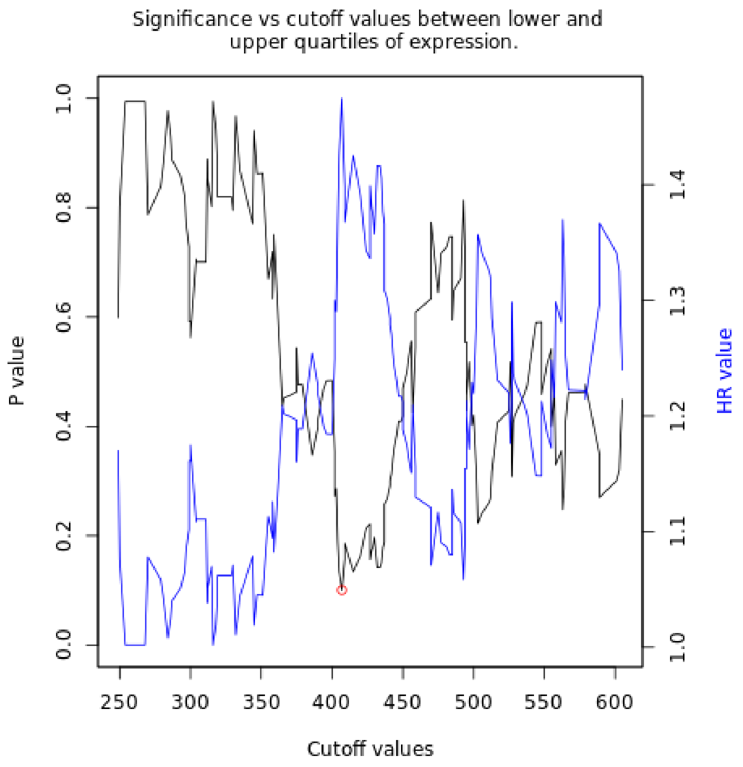

Download p values vs. cutoff table

Median survival

Low expression cohort (months)	High expression cohort (months)
95.3	136.2

Click here for a permanent link

**RNAseq ID:**	CLSPN -
**Survival:**	OS
**Auto select best cutoff:**	checked
**Follow up threshold:**	all
**Censore at threshold:**	checked
**Compute median over entire database:**	false
**Cutoff value used in analysis:**	422
**Expression range of the probe:**	171 - 1548
**Invert HR values below 1:**	not checked

Restrictions

Tumor type: Esophageal Adenocarcinoma

Restrict analysis to subtypes...

Stage:	all
Gender:	all
Race:	all
Grade:	all
Mutation burden:	all
Neoantigen load:	all

Restrict analysis based on cellular content...

Basophils:	all
B-cells:	all
CD4+ memory T-cells:	all
CD8+ T-cells:	all
Eosinophils:	all
Macrophages:	all
Mesenchymal stem cells:	all
Natural killer T-cells:	all
Regulatory T-cells:	all
Type 1 T-helper cells:	all
Type 2 T-helper cells:	all

Results

**P value:**	0.0467
**FDR:**	over 50%

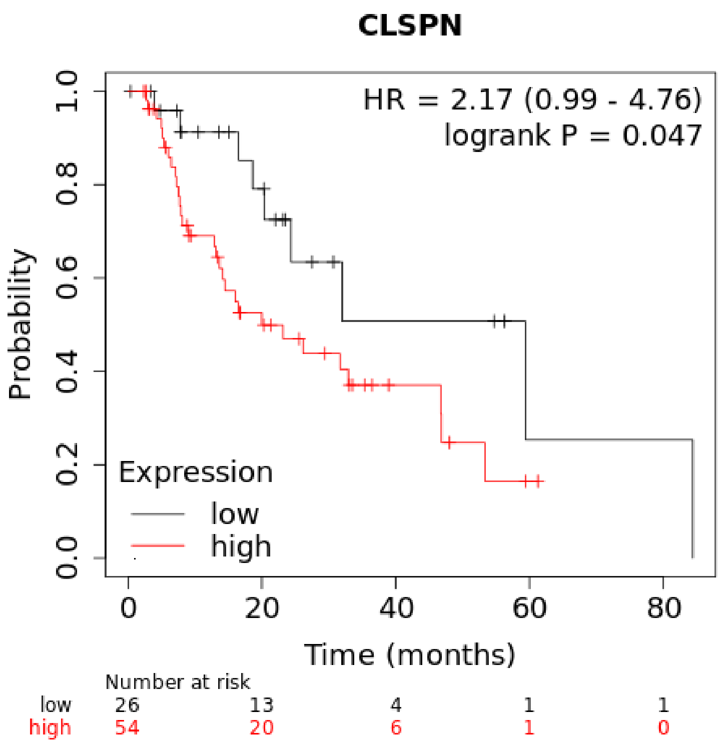

Download plot as a PDF

Auto cutoff plot

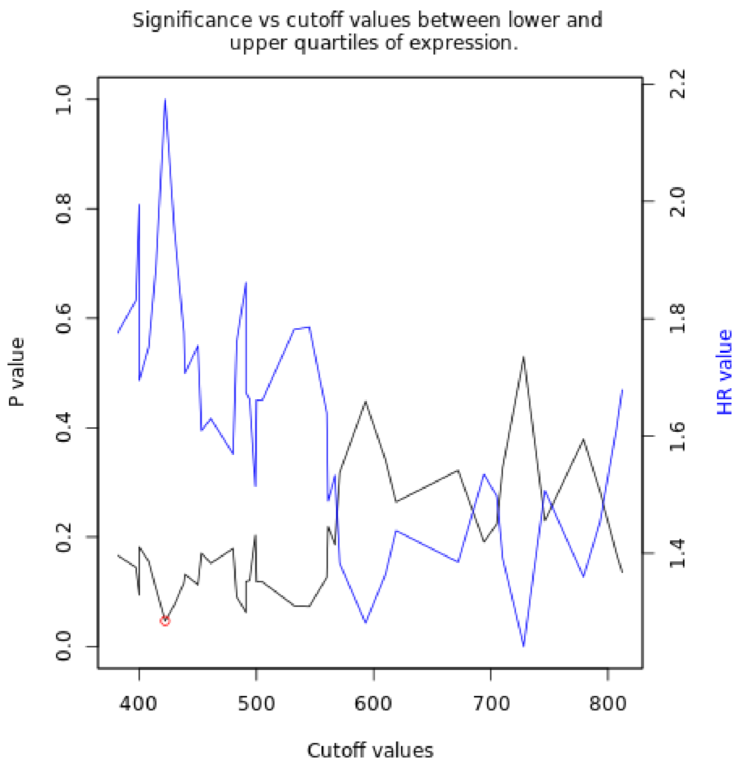

Download p values vs. cutoff table

Median survival

Low expression cohort (months)	High expression cohort (months)
59.37	20

Click here for a permanent link

RNAseq ID: CLSPN -
Survival: OS

**Auto select best cutoff:**	checked
**Follow up threshold:**	all
**Censore at threshold:**	checked
**Compute median over entire database:**	false
**Cutoff value used in analysis:**	599
**Expression range of the probe:**	72 - 1513
**Invert HR values below 1:**	not checked

Restrictions

Tumor type: Esophageal Squamous Cell Carcinoma

Restrict analysis to subtypes...

Stage:	all
Gender:	all
Race:	all
Grade:	all
Mutation burden:	all
Neoantigen load:	all

Restrict analysis based on cellular content...

Basophils:	all
B-cells:	all
CD4+ memory T-cells:	all
CD8+ T-cells:	all
Eosinophils:	all
Macrophages:	all
Mesenchymal stem cells:	all
Natural killer T-cells:	all
Regulatory T-cells:	all
Type 1 T-helper cells:	all
Type 2 T-helper cells:	all

Results

P value: 0.0126
FDR: 50%

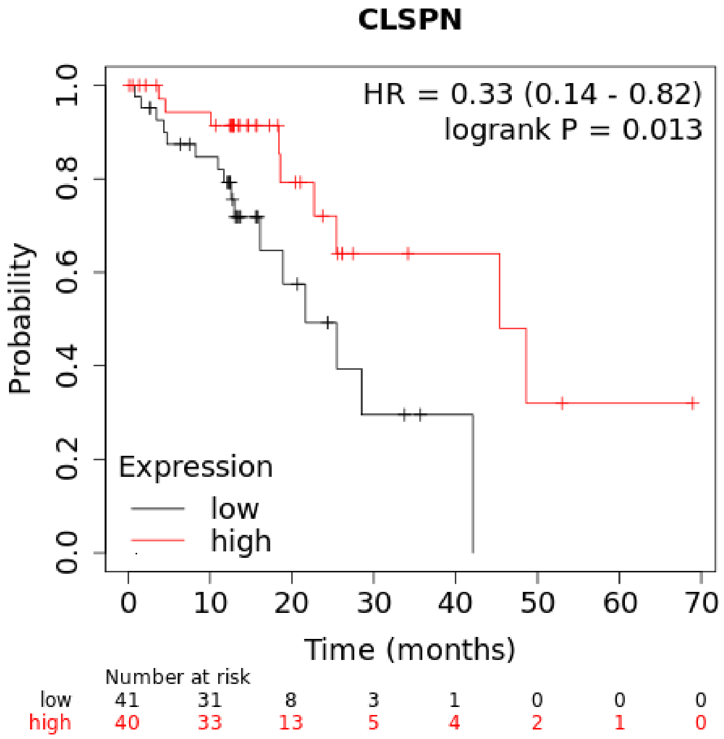

Download plot as a PDF

Auto cutoff plot

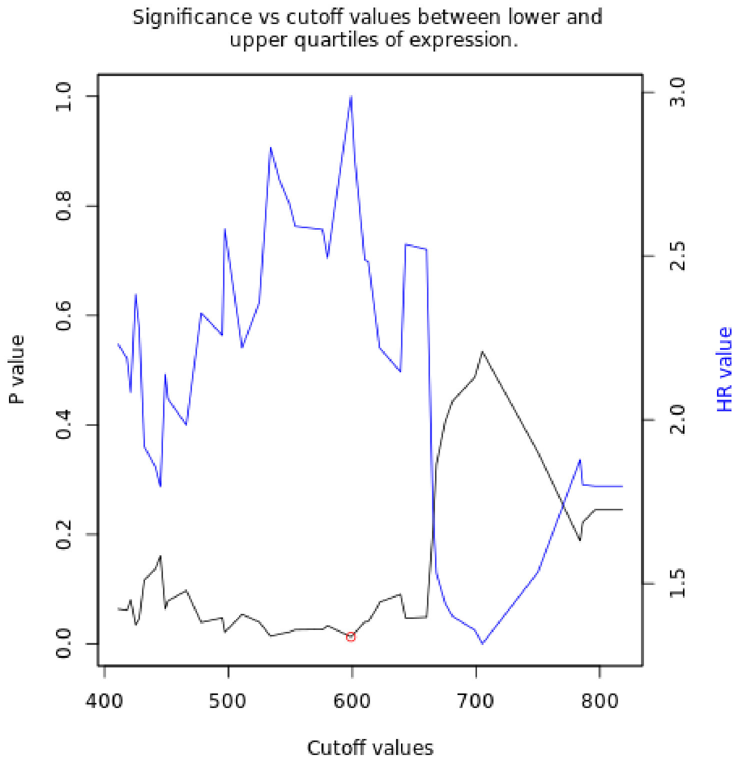

Download p values vs. cutoff table

Median survival

Low expression cohort (months)	High expression cohort (months)
21.67	45.37

Click here for a permanent link

RNAseq ID: CLSPN -
Survival: OS
Auto select best cutoff: checked
Follow up threshold: all
Censore at threshold: checked
Compute median over entire database: false
Cutoff value used in analysis: 249
Expression range of the probe: 14 - 1590
Invert HR values below 1: not checked

Restrictions

Tumor type: Head-neck squamous cell carcinoma

Restrict analysis to subtypes...

Stage: all
Gender: all
Race: all
Grade: all
Mutation burden: all
Neoantigen load: all

Restrict analysis based on cellular content...

Basophils: all
B-cells: all
CD4+ memory T-cells: all
CD8+ T-cells: all

Eosinophils:	all
Macrophages:	all
Mesenchymal stem cells:	all
Natural killer T-cells:	all
Regulatory T-cells:	all
Type 1 T-helper cells:	all
Type 2 T-helper cells:	all

Results

P value: 0.0158
FDR: over 50%

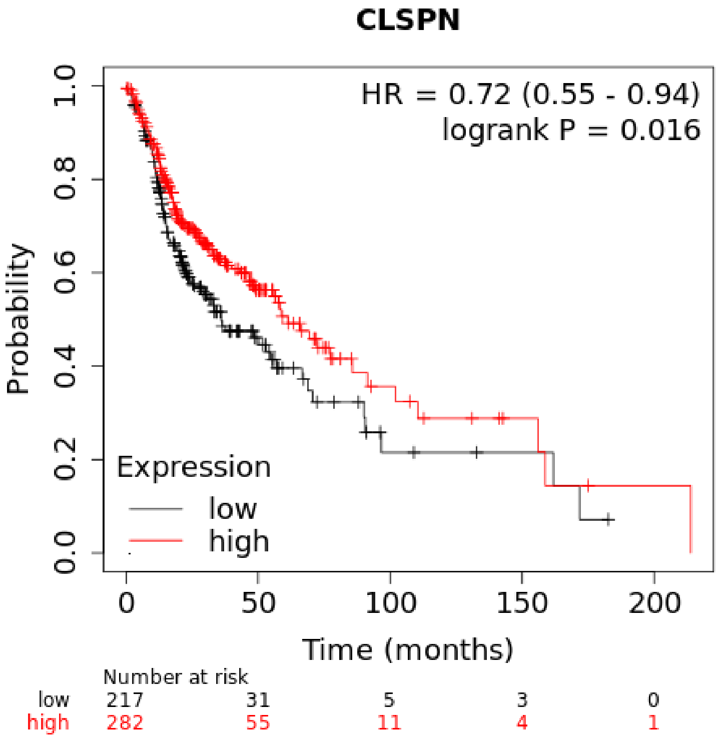

Download plot as a PDF

Auto cutoff plot

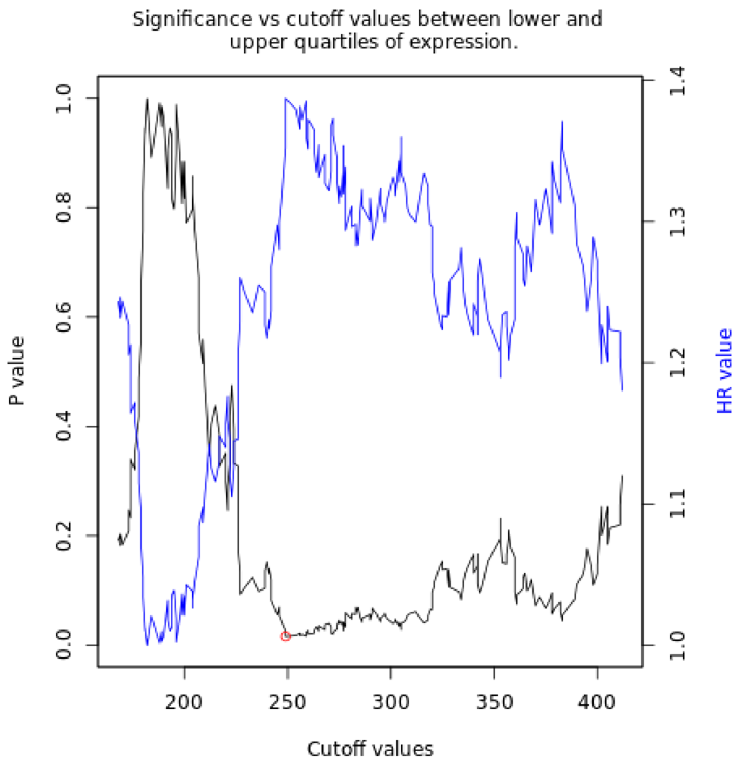

Download p values vs. cutoff table

Median survival

Low expression cohort (months)	High expression cohort (months)
36.03	61.27

Click here for a permanent link

**RNAseq ID:**	CLSPN -
**Survival:**	OS
**Auto select best cutoff:**	checked
**Follow up threshold:**	all
**Censore at threshold:**	checked
**Compute median over entire database:**	false
**Cutoff value used in analysis:**	30
**Expression range of the probe:**	0 - 629
**Invert HR values below 1:**	not checked

Restrictions

Tumor type: Kidney renal clear cell carcinoma

Restrict analysis to subtypes...

Stage:	all
Gender:	all
Race:	all
Grade:	all
Mutation burden:	all
Neoantigen load:	all

Restrict analysis based on cellular content...

Basophils:	all
B-cells:	all
CD4+ memory T-cells:	all
CD8+ T-cells:	all
Eosinophils:	all
Macrophages:	all
Mesenchymal stem cells:	all
Natural killer T-cells:	all
Regulatory T-cells:	all
Type 1 T-helper cells:	all
Type 2 T-helper cells:	all

Results

P value: 0.1552
FDR: 100%

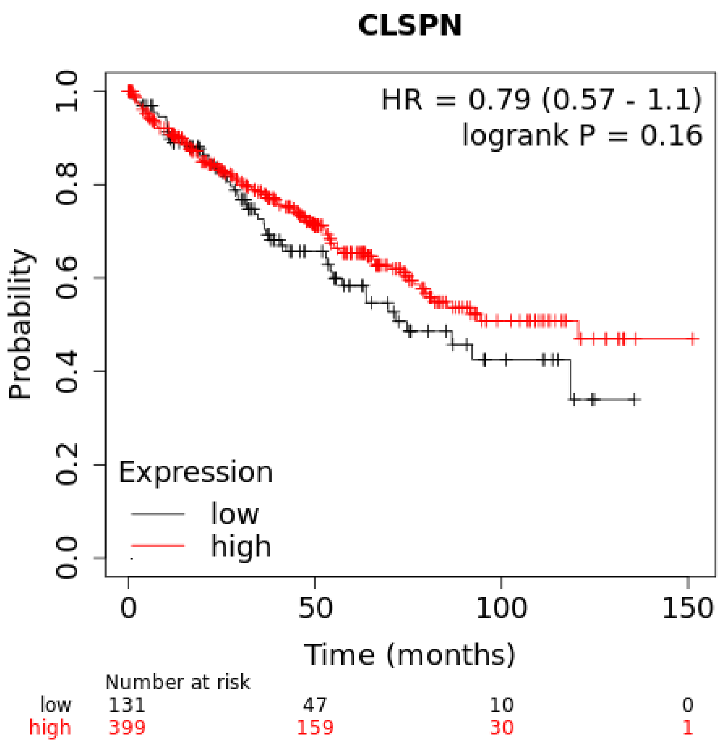

Download plot as a PDF

Auto cutoff plot

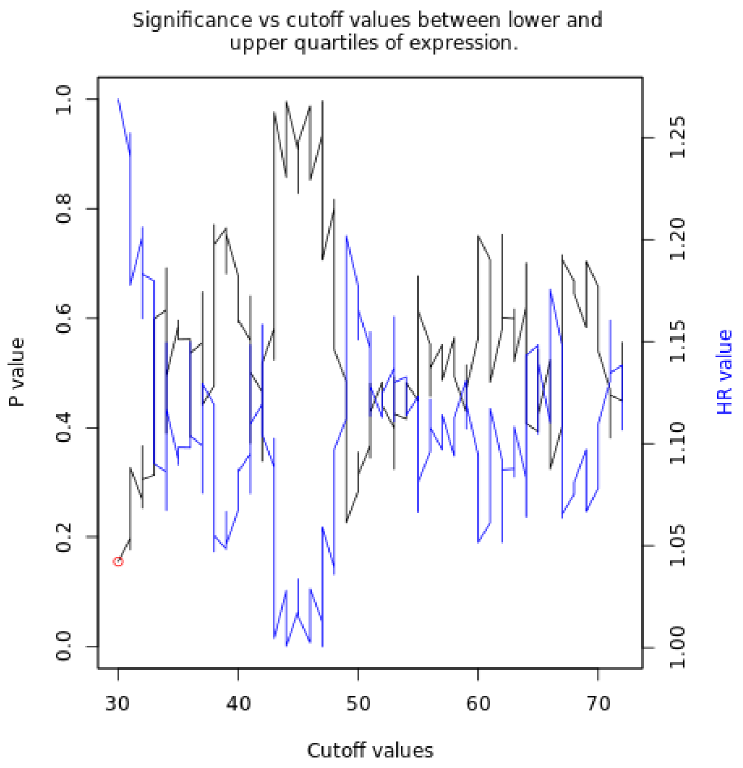

Download p values vs. cutoff table

Median survival

Low expression cohort (months)	High expression cohort (months)
74.7	120.5

Click here for a permanent link

RNAseq ID: CLSPN -
Survival: OS

**Auto select best cutoff:**	checked
**Follow up threshold:**	all
**Censore at threshold:**	checked
**Compute median over entire database:**	false
**Cutoff value used in analysis:**	42
**Expression range of the probe:**	0 - 281
**Invert HR values below 1:**	not checked

Restrictions

Tumor type: Kidney renal papillary cell carcinoma

Restrict analysis to subtypes...

Stage:	all
Gender:	all
Race:	all
Grade:	all
Mutation burden:	all
Neoantigen load:	all

Restrict analysis based on cellular content...

Basophils:	all
B-cells:	all
CD4+ memory T-cells:	all
CD8+ T-cells:	all
Eosinophils:	all
Macrophages:	all
Mesenchymal stem cells:	all
Natural killer T-cells:	all
Regulatory T-cells:	all
Type 1 T-helper cells:	all
Type 2 T-helper cells:	all

Results

P value: 2.3e-6
FDR: 1%

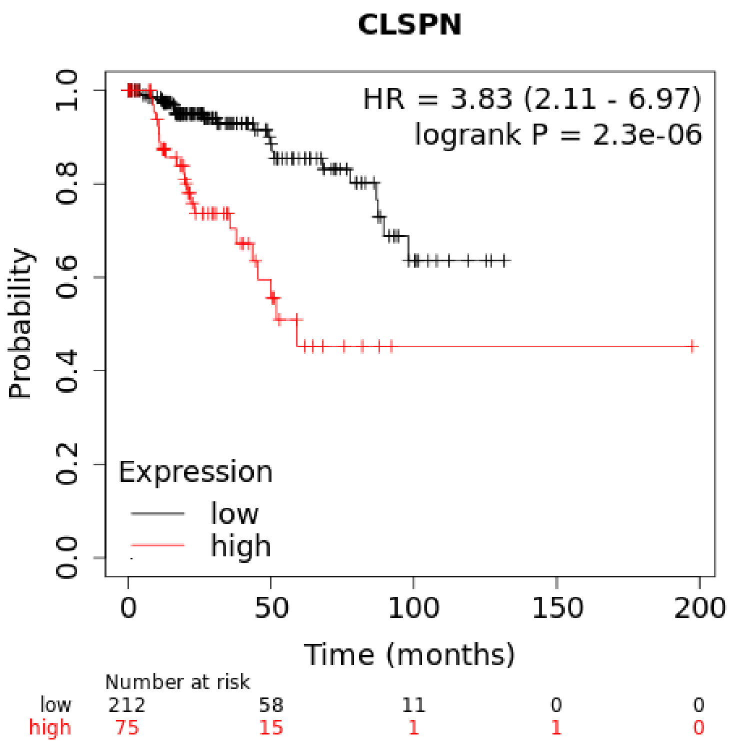

Download plot as a PDF

Auto cutoff plot

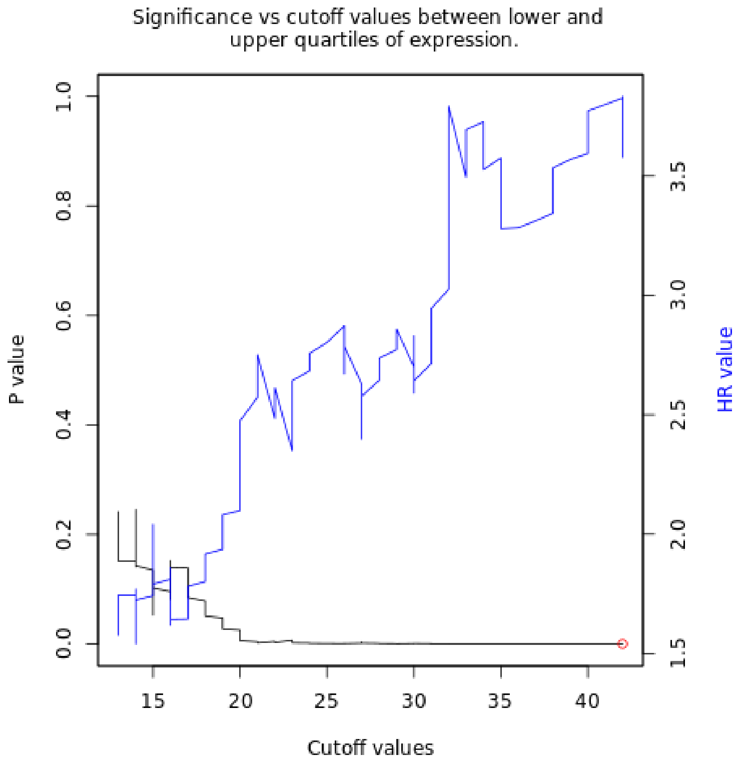

Download p values vs. cutoff table

Upper quartile survival

Low expression cohort (months)	High expression cohort (months)
87.47	23.23

Click here for a permanent link

**RNAseq ID:**	CLSPN -
**Survival:**	OS
**Auto select best cutoff:**	checked
**Follow up threshold:**	all
**Censore at threshold:**	checked
**Compute median over entire database:**	false
**Cutoff value used in analysis:**	32
**Expression range of the probe:**	0 - 527
**Invert HR values below 1:**	not checked

Restrictions

Tumor type: Liver hepatocellular carcinoma

Restrict analysis to subtypes...

Stage:	all
Gender:	all
Race:	all
Grade:	all
Mutation burden:	all
Neoantigen load:	all

Restrict analysis based on cellular content...

Basophils:	all
B-cells:	all
CD4+ memory T-cells:	all
CD8+ T-cells:	all

Eosinophils:	all
Macrophages:	all
Mesenchymal stem cells:	all
Natural killer T-cells:	all
Regulatory T-cells:	all
Type 1 T-helper cells:	all
Type 2 T-helper cells:	all

Results

P value: 1.3e-5
FDR: 1%

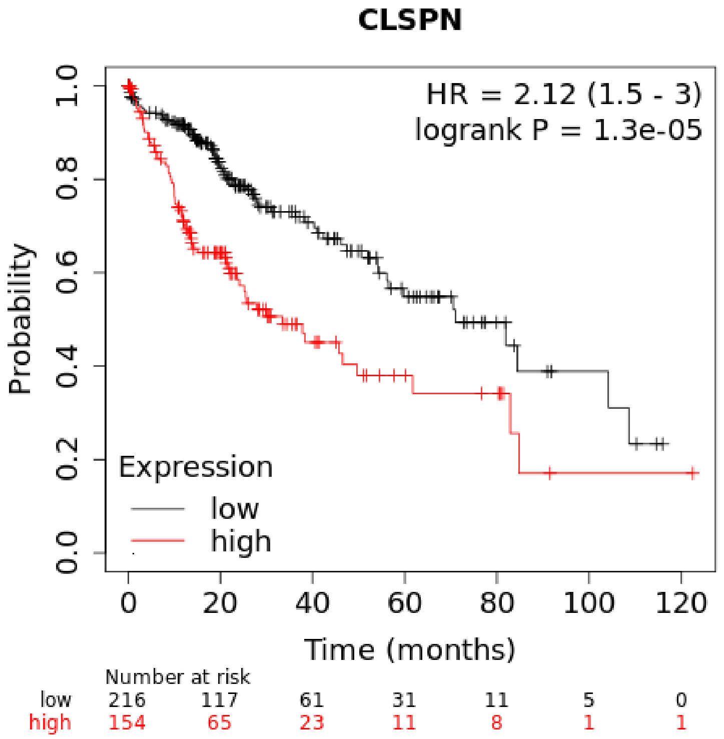

Download plot as a PDF

Auto cutoff plot

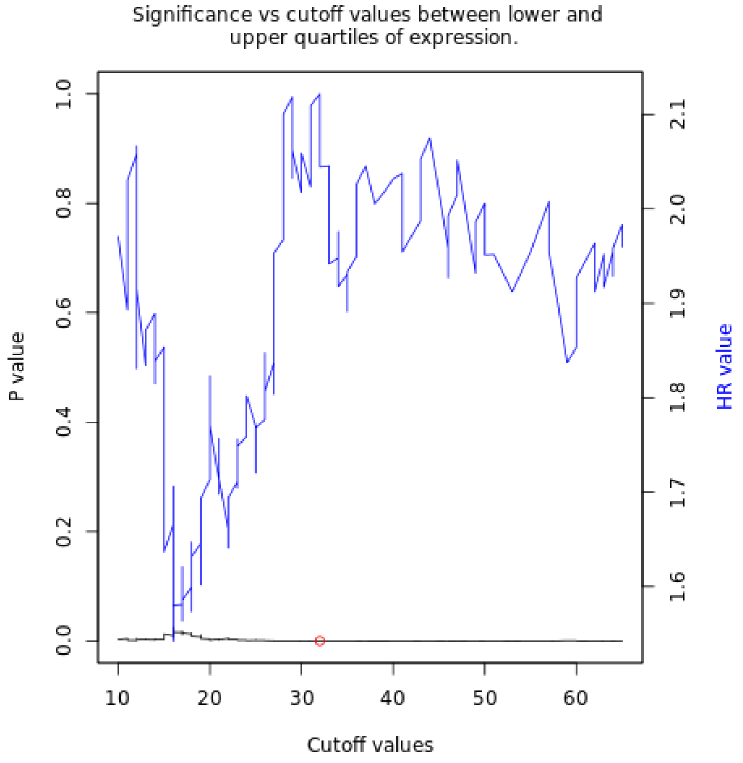

Download p values vs. cutoff table

Median survival

Low expression cohort (months)	High expression cohort (months)
71.03	33.5

Click here for a permanent link

**RNAseq ID:**	CLSPN -
**Survival:**	OS
**Auto select best cutoff:**	checked
**Follow up threshold:**	all
**Censore at threshold:**	checked
**Compute median over entire database:**	false
**Cutoff value used in analysis:**	70
**Expression range of the probe:**	4 - 2159
**Invert HR values below 1:**	not checked

Restrictions

Tumor type: Lung adenocarcinoma

Restrict analysis to subtypes...

Stage:	all
Gender:	all
Race:	all
Grade:	all
Mutation burden:	all
Neoantigen load:	all

Restrict analysis based on cellular content...

Basophils:	all
B-cells:	all
CD4+ memory T-cells:	all
CD8+ T-cells:	all
Eosinophils:	all
Macrophages:	all
Mesenchymal stem cells:	all
Natural killer T-cells:	all
Regulatory T-cells:	all
Type 1 T-helper cells:	all
Type 2 T-helper cells:	all

Results

**P value:**	0.0011
**FDR:**	20%

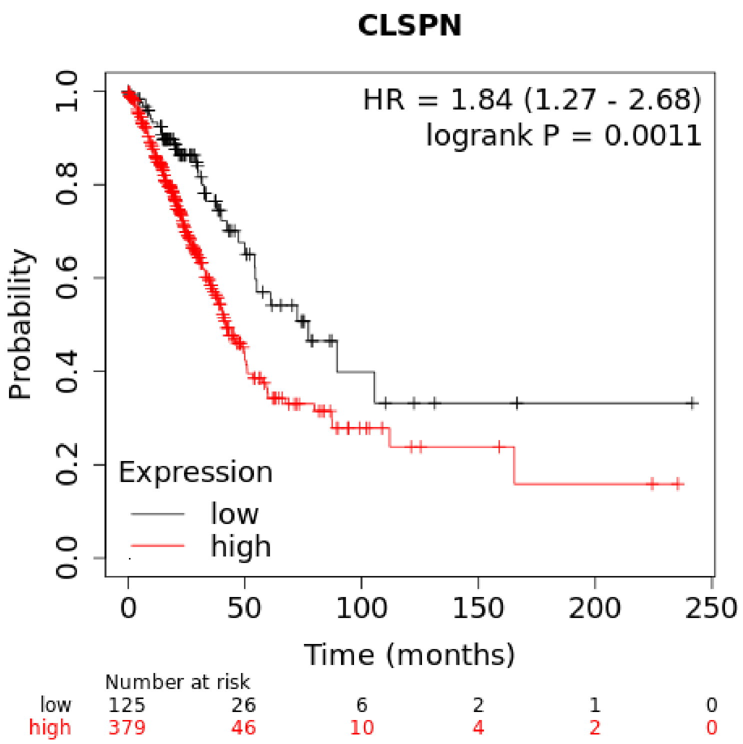

Download plot as a PDF

Auto cutoff plot

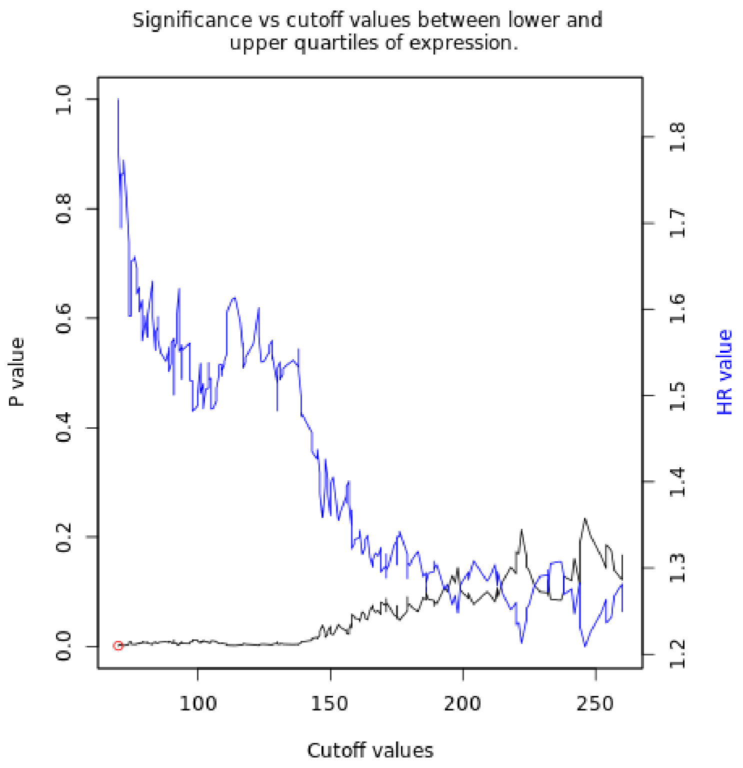

Download p values vs. cutoff table

Median survival

Low expression cohort (months)	High expression cohort (months)
77.27	42.17

Click here for a permanent link

RNAseq ID: CLSPN -

Survival: OS

**Auto select best cutoff:**	checked
**Follow up threshold:**	all
**Censore at threshold:**	checked
**Compute median over entire database:**	false
**Cutoff value used in analysis:**	349
**Expression range of the probe:**	11 - 1890
**Invert HR values below 1:**	not checked

Restrictions

Tumor type: Lung squamous cell carcinoma

Restrict analysis to subtypes...

Stage:	all
Gender:	all
Race:	all
Grade:	all
Mutation burden:	all
Neoantigen load:	all

Restrict analysis based on cellular content...

Basophils:	all
B-cells:	all
CD4+ memory T-cells:	all
CD8+ T-cells:	all
Eosinophils:	all
Macrophages:	all
Mesenchymal stem cells:	all
Natural killer T-cells:	all
Regulatory T-cells:	all
Type 1 T-helper cells:	all
Type 2 T-helper cells:	all

Results

P value: 0.5182
FDR: 100%

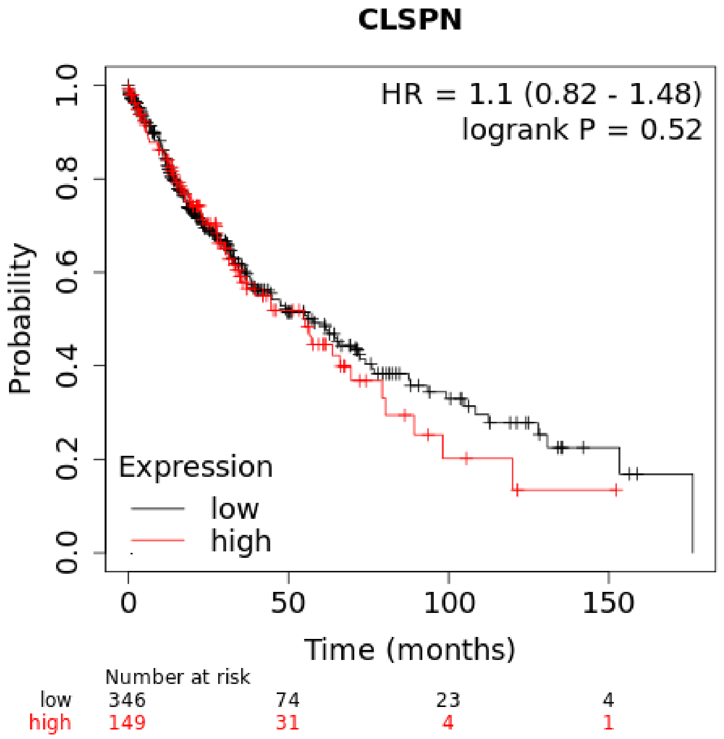

Download plot as a PDF

Auto cutoff plot

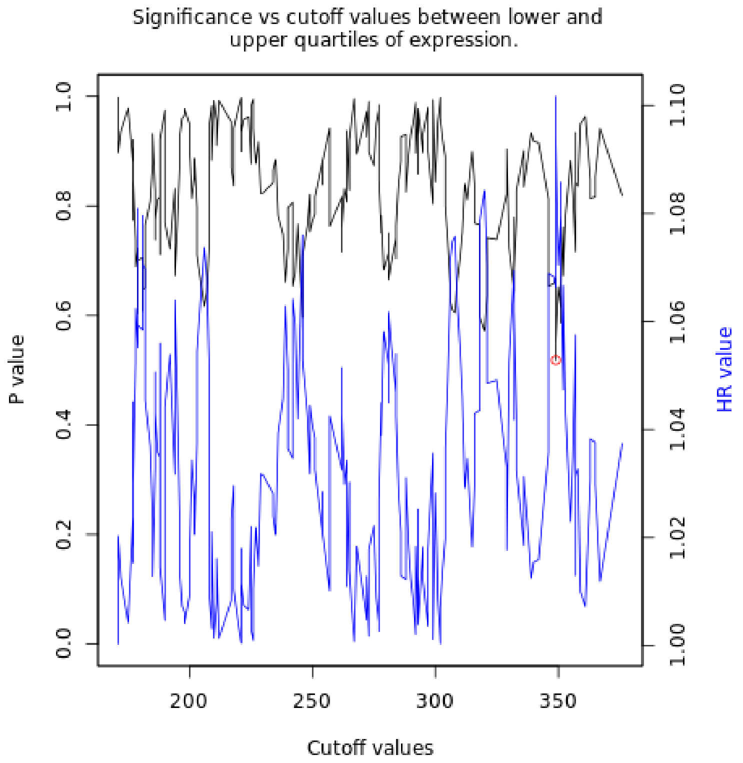

Download p values vs. cutoff table

Median survival

Low expression cohort (months)	High expression cohort (months)
57.87	55.2

Click here for a permanent link

RNAseq ID: CLSPN -
Survival: OS
Auto select best cutoff: checked
Follow up threshold: all
Censore at threshold: checked
Compute median over entire database: false
Cutoff value used in analysis: 145
Expression range of the probe: 9 - 1280
Invert HR values below 1: not checked

Restrictions

Tumor type: Ovarian cancer

Restrict analysis to subtypes...

Stage: all
Gender: all
Race: all
Grade: all
Mutation burden: all
Neoantigen load: all

Restrict analysis based on cellular content...

Basophils: all
B-cells: all
CD4+ memory T-cells: all
CD8+ T-cells: all

Eosinophils: all
Macrophages: all
Mesenchymal stem cells: all
Natural killer T-cells: all
Regulatory T-cells: all
Type 1 T-helper cells: all
Type 2 T-helper cells: all

Results

P value: 0.0166
FDR: over 50%

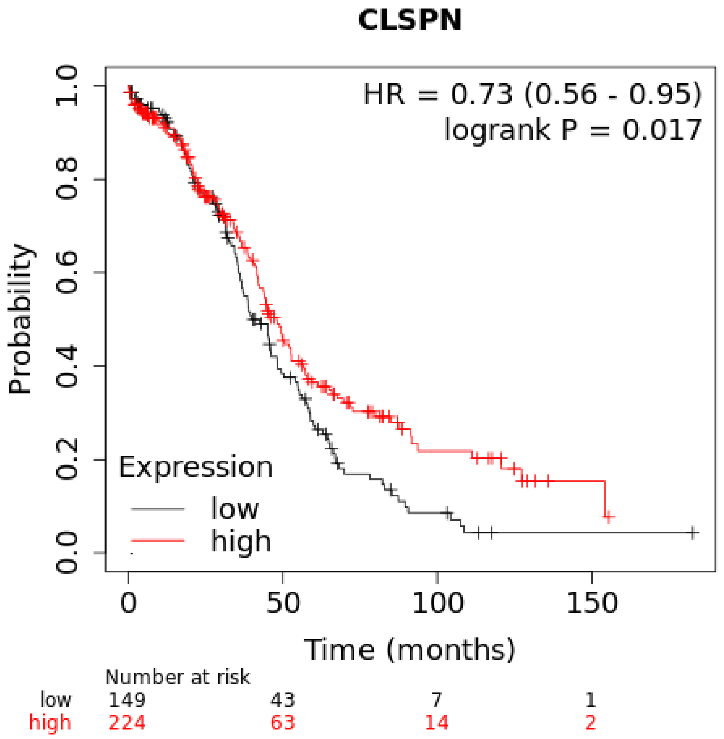

Download plot as a PDF

Auto cutoff plot

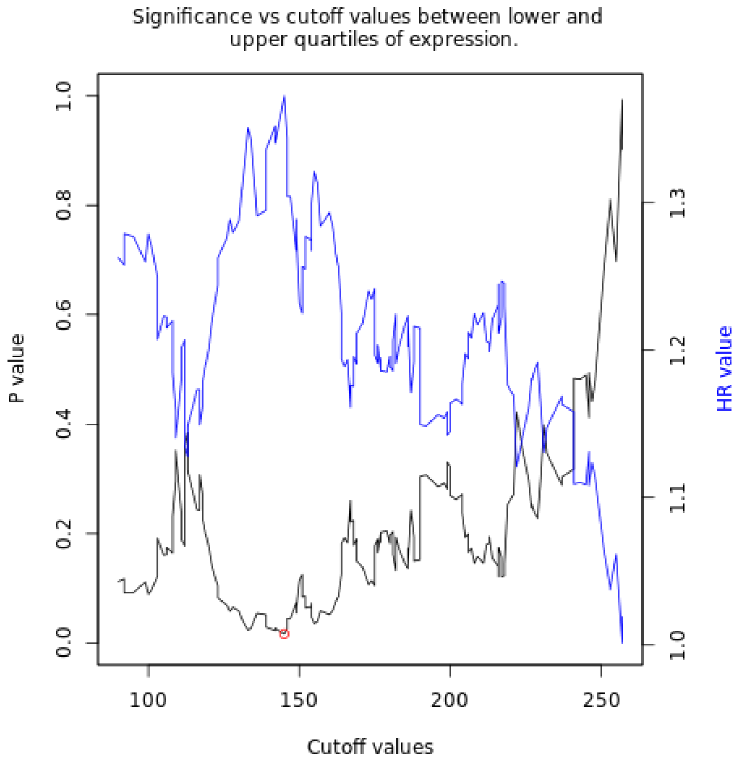

Download p values vs. cutoff table

Median survival

Low expression cohort (months)	High expression cohort (months)
39.97	48.07

Click here for a permanent link

**RNAseq ID:**	CLSPN -
**Survival:**	OS
**Auto select best cutoff:**	checked
**Follow up threshold:**	all
**Censore at threshold:**	checked
**Compute median over entire database:**	false
**Cutoff value used in analysis:**	72
**Expression range of the probe:**	2 - 352
**Invert HR values below 1:**	not checked

Restrictions

Tumor type: Pancreatic ductal adenocarcinoma

Restrict analysis to subtypes...

Stage:	all
Gender:	all
Race:	all
Grade:	all
Mutation burden:	all
Neoantigen load:	all

Restrict analysis based on cellular content...

Basophils:	all
B-cells:	all
CD4+ memory T-cells:	all
CD8+ T-cells:	all
Eosinophils:	all
Macrophages:	all
Mesenchymal stem cells:	all
Natural killer T-cells:	all
Regulatory T-cells:	all
Type 1 T-helper cells:	all
Type 2 T-helper cells:	all

Results

P value: 8.5e-6
FDR: 1%

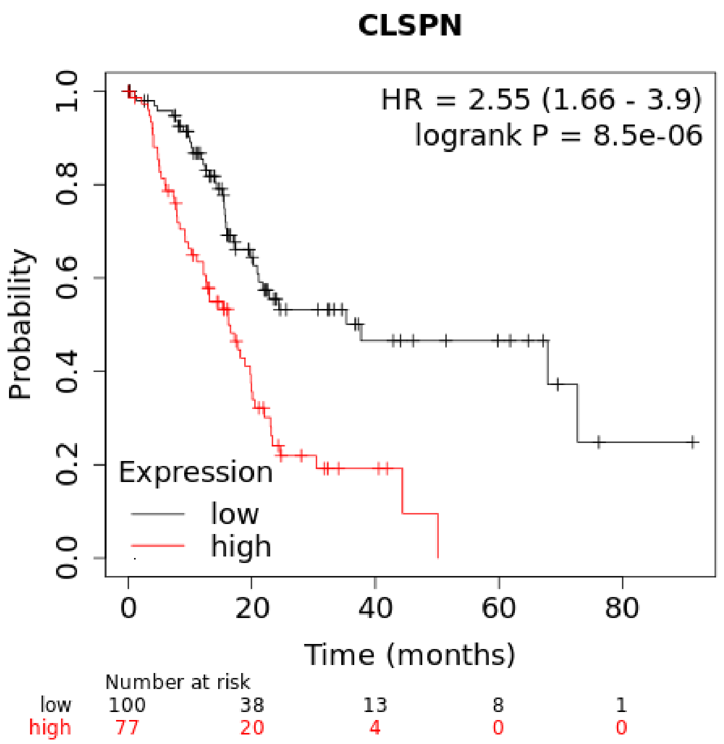

Download plot as a PDF

Auto cutoff plot

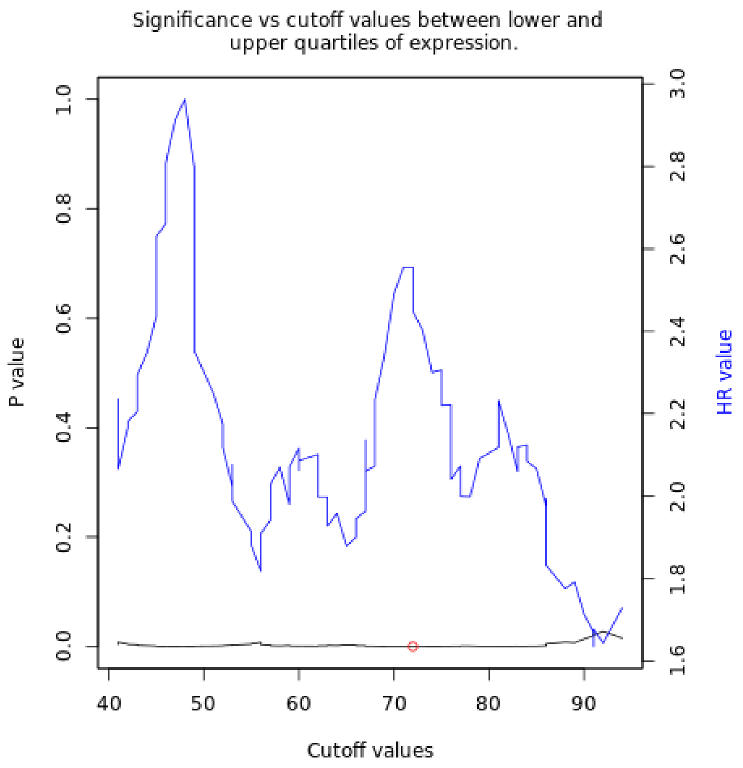

Download p values vs. cutoff table

Median survival

Low expression cohort (months)	High expression cohort (months)
37.67	16.2

Click here for a permanent link

RNAseq ID: CLSPN -
Survival: OS

**Auto select best cutoff:**	checked
**Follow up threshold:**	all
**Censore at threshold:**	checked
**Compute median over entire database:**	false
**Cutoff value used in analysis:**	13
**Expression range of the probe:**	0 - 155
**Invert HR values below 1:**	not checked

Restrictions

Tumor type: Pheochromocytoma and Paraganglioma

Restrict analysis to subtypes...

Stage:	all
Gender:	all
Race:	all
Grade:	all
Mutation burden:	all
Neoantigen load:	all

Restrict analysis based on cellular content...

Basophils:	all
B-cells:	all
CD4+ memory T-cells:	all
CD8+ T-cells:	all
Eosinophils:	all
Macrophages:	all
Mesenchymal stem cells:	all
Natural killer T-cells:	all
Regulatory T-cells:	all
Type 1 T-helper cells:	all
Type 2 T-helper cells:	all

Results

P value: 0.0265
FDR: over 50%

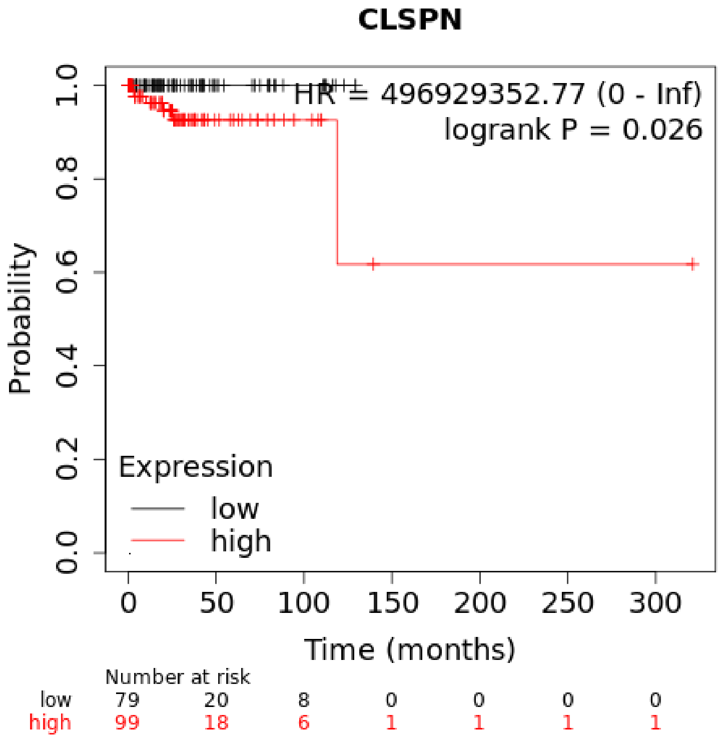

Download plot as a PDF

Auto cutoff plot

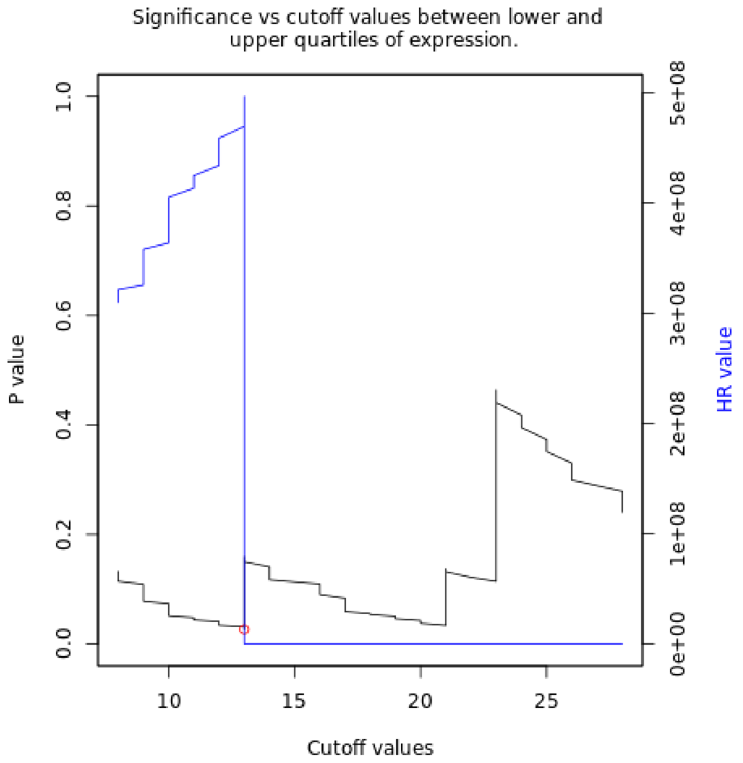

Download p values vs. cutoff table

Median survival

Low expression cohort (months)	High expression cohort (months)
NA	NA

Click here for a permanent link

**RNAseq ID:**	CLSPN	-
**Survival:**	OS	
**Auto select best cutoff:**	checked	
**Follow up threshold:**	all	
**Censore at threshold:**	checked	
**Compute median over entire database:**	false	
**Cutoff value used in analysis:**	168	
**Expression range of the probe:**	39 - 858	
**Invert HR values below 1:**	not checked	

Restrictions

Tumor type: Rectum adenocarcinoma

Restrict analysis to subtypes...

Stage:	all
Gender:	all
Race:	all
Grade:	all
Mutation burden:	all
Neoantigen load:	all

Restrict analysis based on cellular content...

Basophils:	all
B-cells:	all
CD4+ memory T-cells:	all
CD8+ T-cells:	all

Eosinophils:	all
Macrophages:	all
Mesenchymal stem cells:	all
Natural killer T-cells:	all
Regulatory T-cells:	all
Type 1 T-helper cells:	all
Type 2 T-helper cells:	all

Results

P value: 0.0108
FDR: over 50%

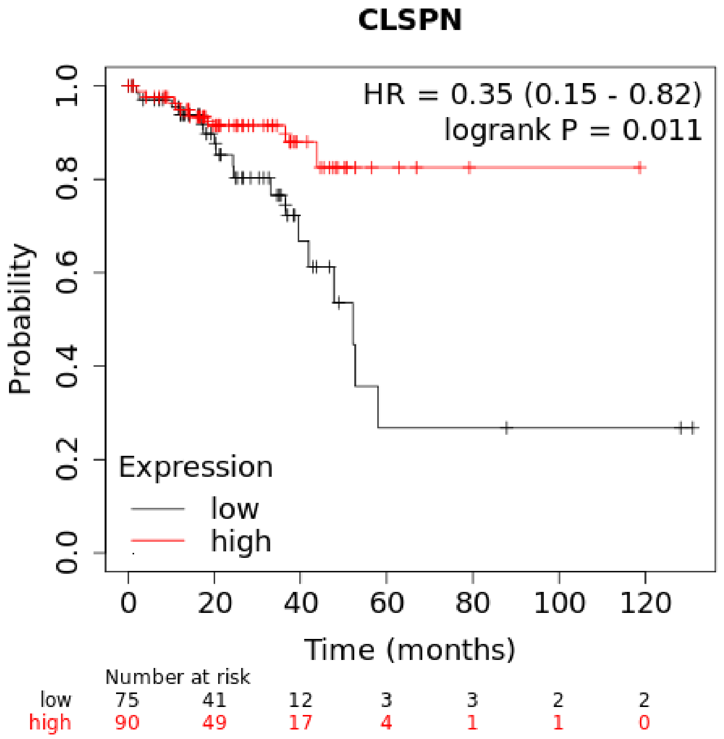

Download plot as a PDF

Auto cutoff plot

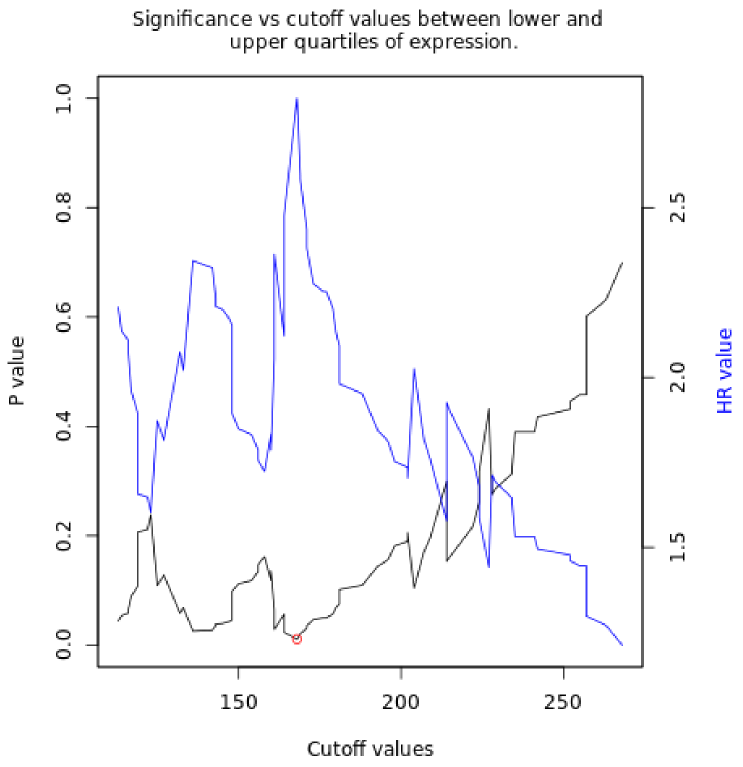

Download p values vs. cutoff table

Median survival

Low expression cohort (months)	High expression cohort (months)
NA	NA

Click here for a permanent link

**RNAseq ID:**	CLSPN -
**Survival:**	OS
**Auto select best cutoff:**	checked
**Follow up threshold:**	all
**Censore at threshold:**	checked
**Compute median over entire database:**	false
**Cutoff value used in analysis:**	256
**Expression range of the probe:**	4 - 1745
**Invert HR values below 1:**	not checked

Restrictions

Tumor type: Sarcoma

Restrict analysis to subtypes...

Stage:	all
Gender:	all
Race:	all
Grade:	all
Mutation burden:	all
Neoantigen load:	all

Restrict analysis based on cellular content...

Basophils:	all
B-cells:	all
CD4+ memory T-cells:	all
CD8+ T-cells:	all
Eosinophils:	all
Macrophages:	all
Mesenchymal stem cells:	all
Natural killer T-cells:	all
Regulatory T-cells:	all
Type 1 T-helper cells:	all
Type 2 T-helper cells:	all

Results

**P value:**	0.0015
**FDR:**	20%

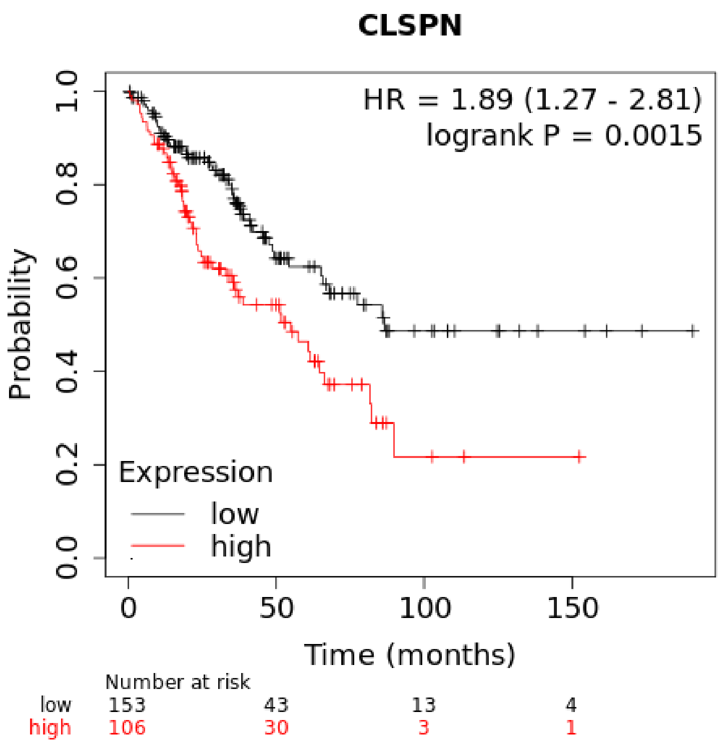

Download plot as a PDF

Auto cutoff plot

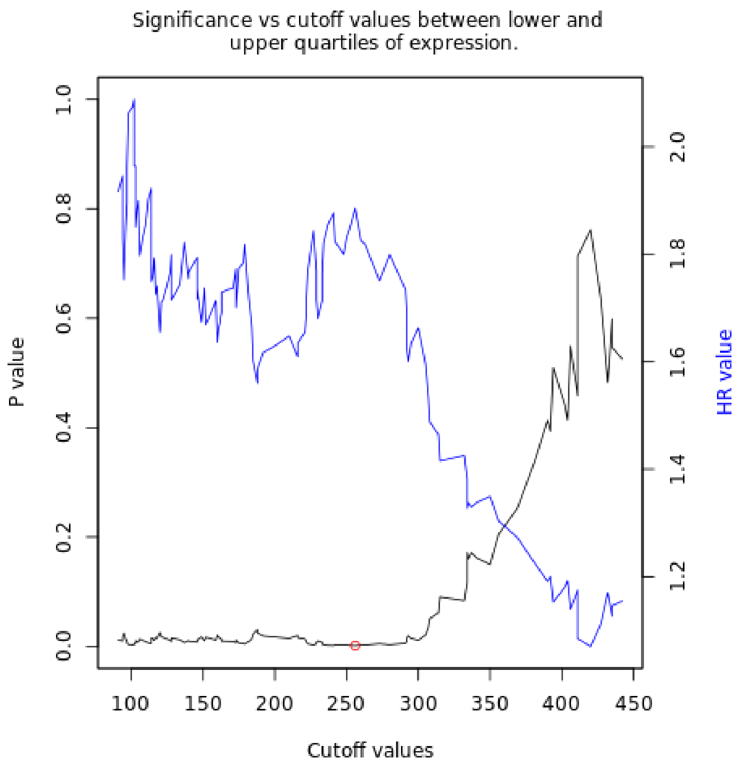

Download p values vs. cutoff table

Median survival

Low expression cohort (months)	High expression cohort (months)
86.63	54.97

Click here for a permanent link

RNAseq ID: CLSPN -
Survival: OS

**Auto select best cutoff:**	checked
**Follow up threshold:**	all
**Censore at threshold:**	checked
**Compute median over entire database:**	false
**Cutoff value used in analysis:**	457
**Expression range of the probe:**	6 - 1560
**Invert HR values below 1:**	not checked

Restrictions

Tumor type: Stomach adenocarcinoma

Restrict analysis to subtypes...

Stage:	all
Gender:	all
Race:	all
Grade:	all
Mutation burden:	all
Neoantigen load:	all

Restrict analysis based on cellular content...

Basophils:	all
B-cells:	all
CD4+ memory T-cells:	all
CD8+ T-cells:	all
Eosinophils:	all
Macrophages:	all
Mesenchymal stem cells:	all
Natural killer T-cells:	all
Regulatory T-cells:	all
Type 1 T-helper cells:	all
Type 2 T-helper cells:	all

Results

P value: 0.0004
FDR: 10%

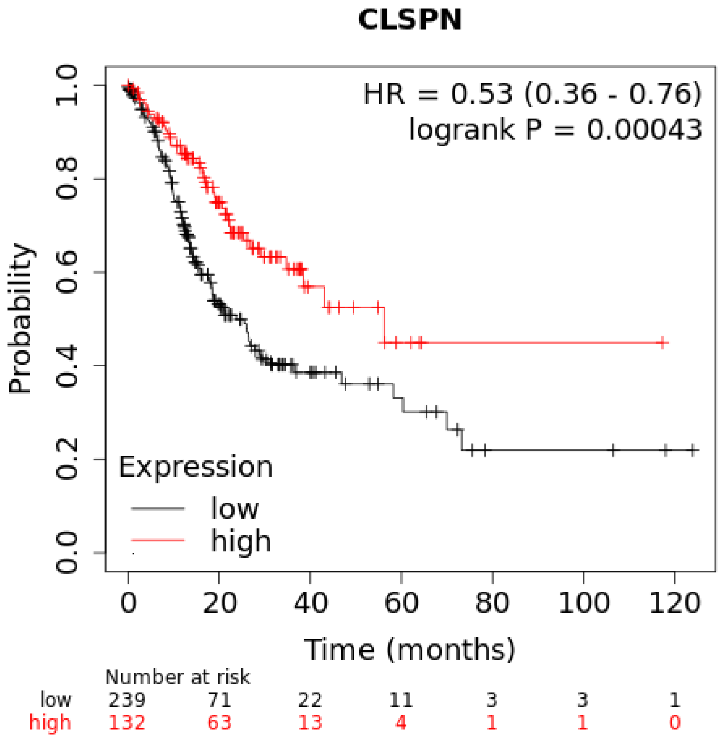

Download plot as a PDF

Auto cutoff plot

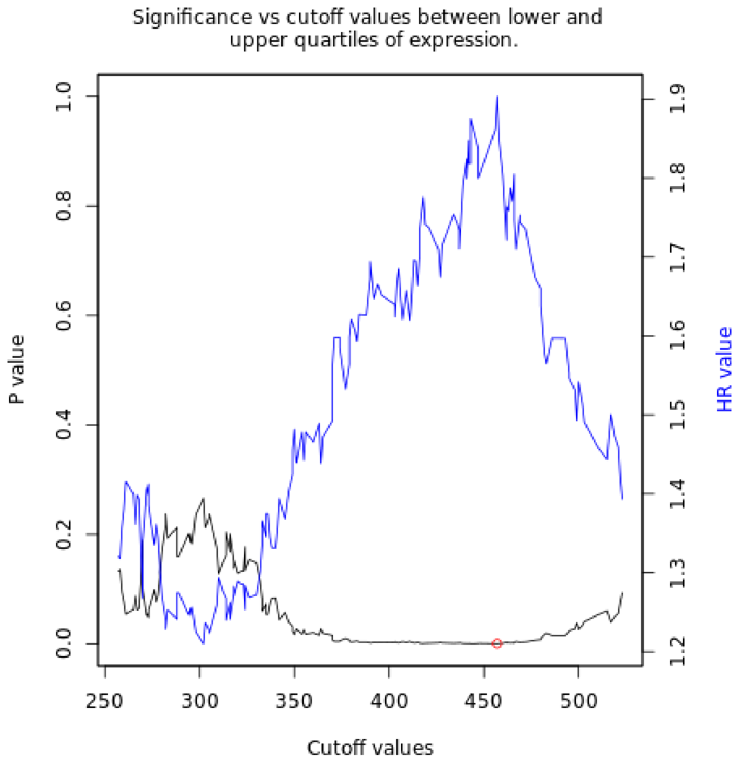

Download p values vs. cutoff table

Median survival

Low expression cohort (months)	High expression cohort (months)
23.73	56.2

Click here for a permanent link

**RNAseq ID:**	CLSPN	-
**Survival:**	OS	
**Auto select best cutoff:**	checked	
**Follow up threshold:**	all	
**Censore at threshold:**	checked	
**Compute median over entire database:**	false	
**Cutoff value used in analysis:**	465	
**Expression range of the probe:**	42 - 1728	
**Invert HR values below 1:**	not checked	

Restrictions

Tumor type: Testicular Germ Cell Tumor

Restrict analysis to subtypes...

Stage:	all
Gender:	all
Race:	all
Grade:	all
Mutation burden:	all
Neoantigen load:	all

Restrict analysis based on cellular content...

Basophils:	all
B-cells:	all
CD4+ memory T-cells:	all
CD8+ T-cells:	all

Eosinophils: all
Macrophages: all
Mesenchymal stem cells: all
Natural killer T-cells: all
Regulatory T-cells: all
Type 1 T-helper cells: all
Type 2 T-helper cells: all

Results

P value: 0.5957
FDR: 100%

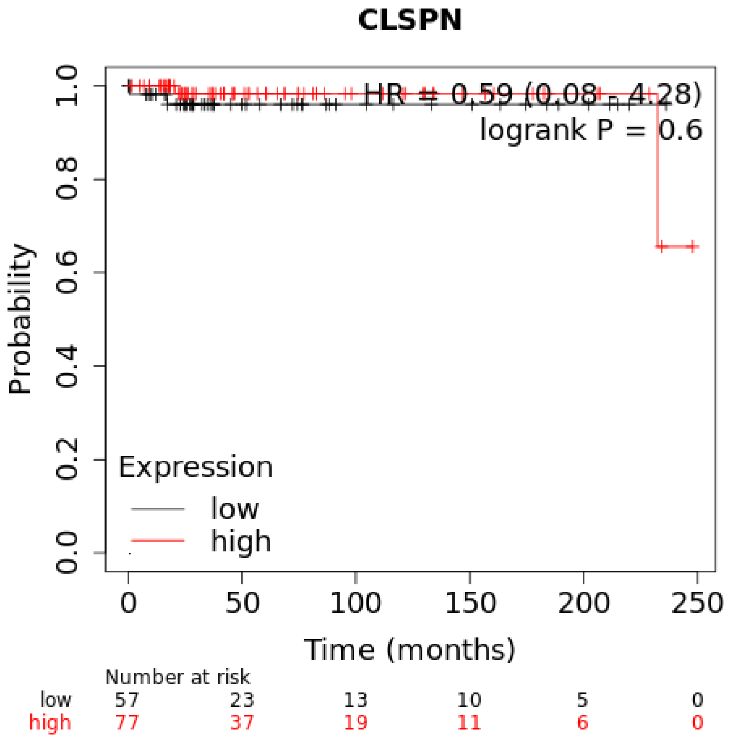

Download plot as a PDF

Auto cutoff plot

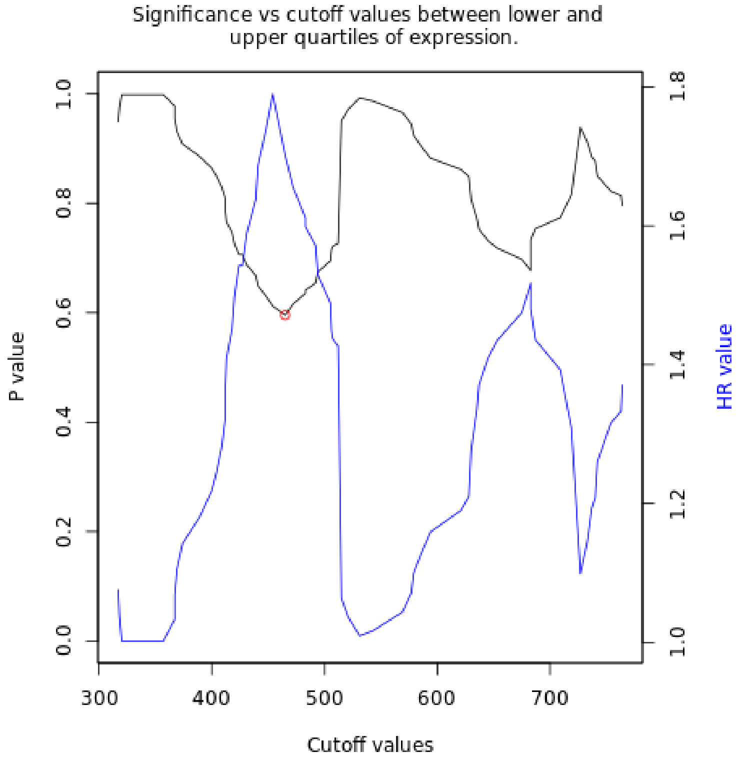

Download p values vs. cutoff table

Median survival

Low expression cohort (months)	High expression cohort (months)
NA	NA

Click here for a permanent link

**RNAseq ID:**	CLSPN -
**Survival:**	OS
**Auto select best cutoff:**	checked
**Follow up threshold:**	all
**Censore at threshold:**	checked
**Compute median over entire database:**	false
**Cutoff value used in analysis:**	133
**Expression range of the probe:**	8 - 891
**Invert HR values below 1:**	not checked

Restrictions

Tumor type: Thymoma

Restrict analysis to subtypes...

Stage:	all
Gender:	all
Race:	all
Grade:	all
Mutation burden:	all
Neoantigen load:	all

Restrict analysis based on cellular content...

Basophils:	all
B-cells:	all
CD4+ memory T-cells:	all
CD8+ T-cells:	all
Eosinophils:	all
Macrophages:	all
Mesenchymal stem cells:	all
Natural killer T-cells:	all
Regulatory T-cells:	all
Type 1 T-helper cells:	all
Type 2 T-helper cells:	all

Results

**P value:**	0.0044
**FDR:**	20%

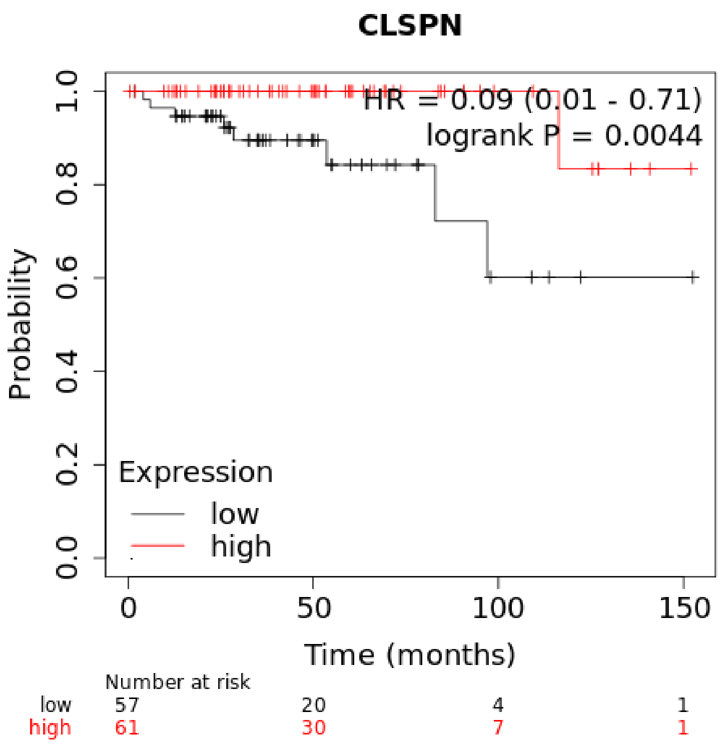

Download plot as a PDF

Auto cutoff plot

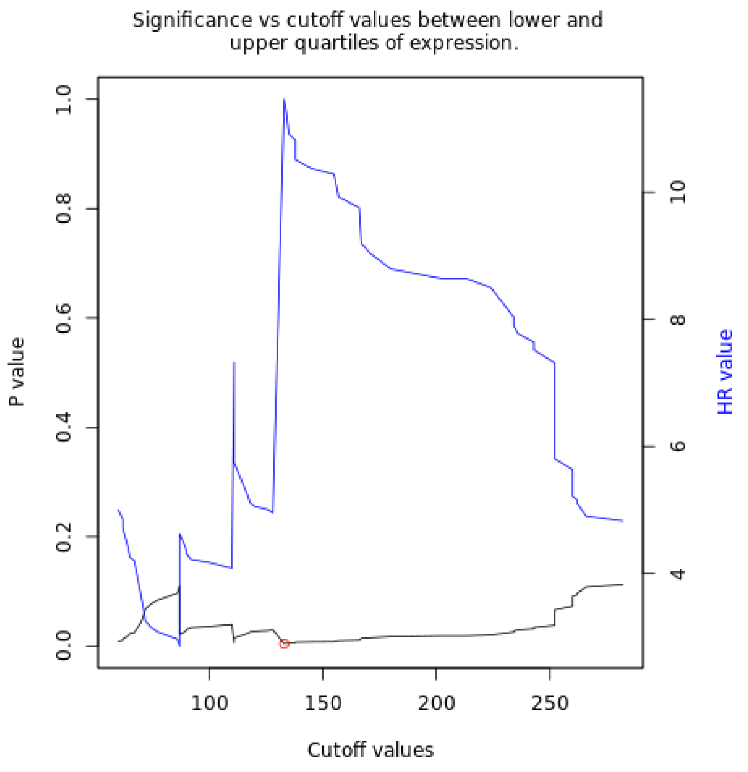

Download p values vs. cutoff table

Median survival

Low expression cohort (months)	High expression cohort (months)
NA	NA

Click here for a permanent link

RNAseq ID: CLSPN -
Survival: OS

**Auto select best cutoff:**	checked
**Follow up threshold:**	all
**Censore at threshold:**	checked
**Compute median over entire database:**	false
**Cutoff value used in analysis:**	24
**Expression range of the probe:**	1 - 197
**Invert HR values below 1:**	not checked

Restrictions

Tumor type: Thyroid carcinoma

Restrict analysis to subtypes...

Stage:	all
Gender:	all
Race:	all
Grade:	all
Mutation burden:	all
Neoantigen load:	all

Restrict analysis based on cellular content...

Basophils:	all
B-cells:	all
CD4+ memory T-cells:	all
CD8+ T-cells:	all
Eosinophils:	all
Macrophages:	all
Mesenchymal stem cells:	all
Natural killer T-cells:	all
Regulatory T-cells:	all
Type 1 T-helper cells:	all
Type 2 T-helper cells:	all

Results

P value: 0.1416
FDR: 100%

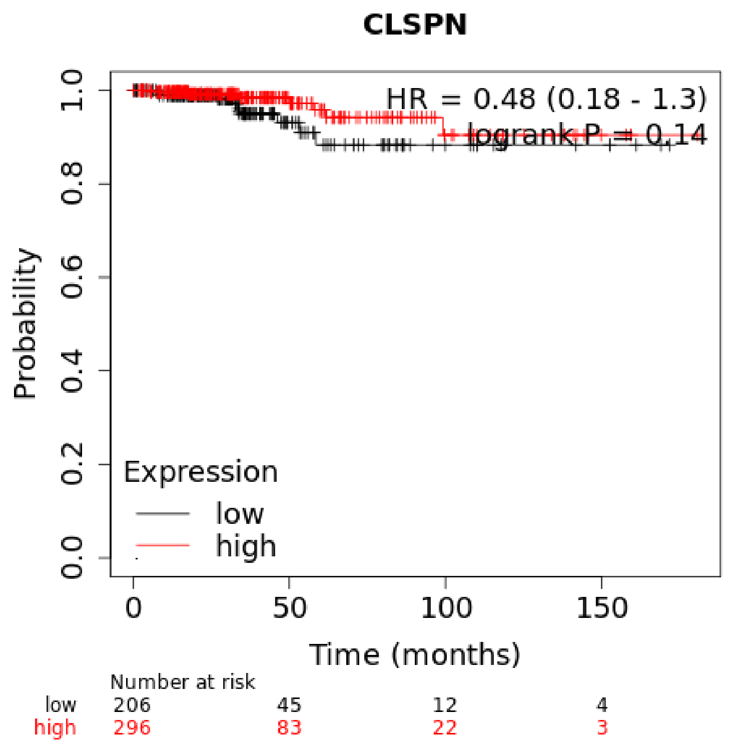

Download plot as a PDF

Auto cutoff plot

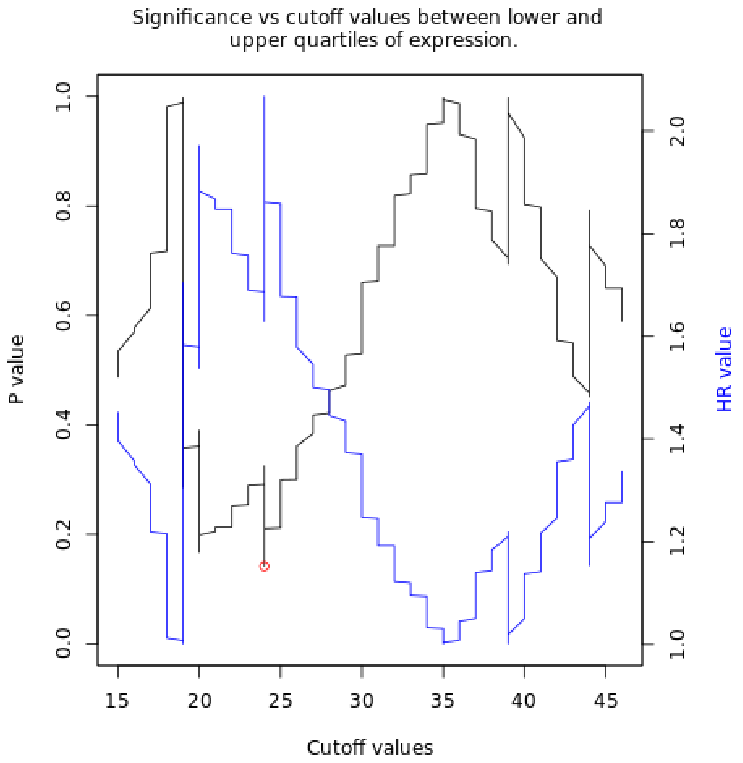

Download p values vs. cutoff table

Median survival

Low expression cohort (months)	High expression cohort (months)
NA	NA

Click here for a permanent link

**RNAseq ID:**	CLSPN -
**Survival:**	OS
**Auto select best cutoff:**	checked
**Follow up threshold:**	all
**Censore at threshold:**	checked
**Compute median over entire database:**	false
**Cutoff value used in analysis:**	88
**Expression range of the probe:**	0 - 1177
**Invert HR values below 1:**	not checked

Restrictions

Tumor type: Uterine corpus endometrial carcinoma

Restrict analysis to subtypes...

Stage:	all
Gender:	all
Race:	all
Grade:	all
Mutation burden:	all
Neoantigen load:	all

Restrict analysis based on cellular content...

Basophils:	all
B-cells:	all
CD4+ memory T-cells:	all
CD8+ T-cells:	all

Eosinophils:	all
Macrophages:	all
Mesenchymal stem cells:	all
Natural killer T-cells:	all
Regulatory T-cells:	all
Type 1 T-helper cells:	all
Type 2 T-helper cells:	all

Results

P value: 0.0354
FDR: over 50%

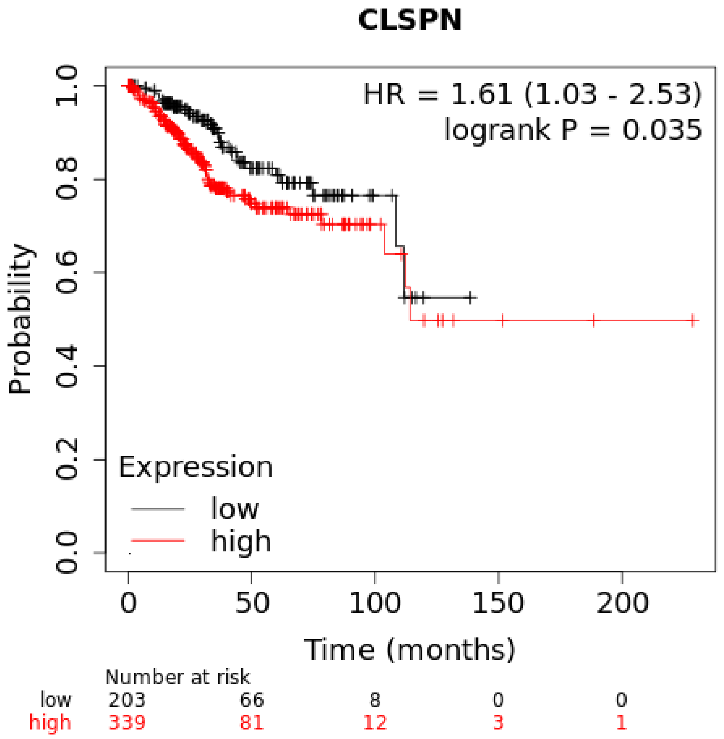

Download plot as a PDF

Auto cutoff plot

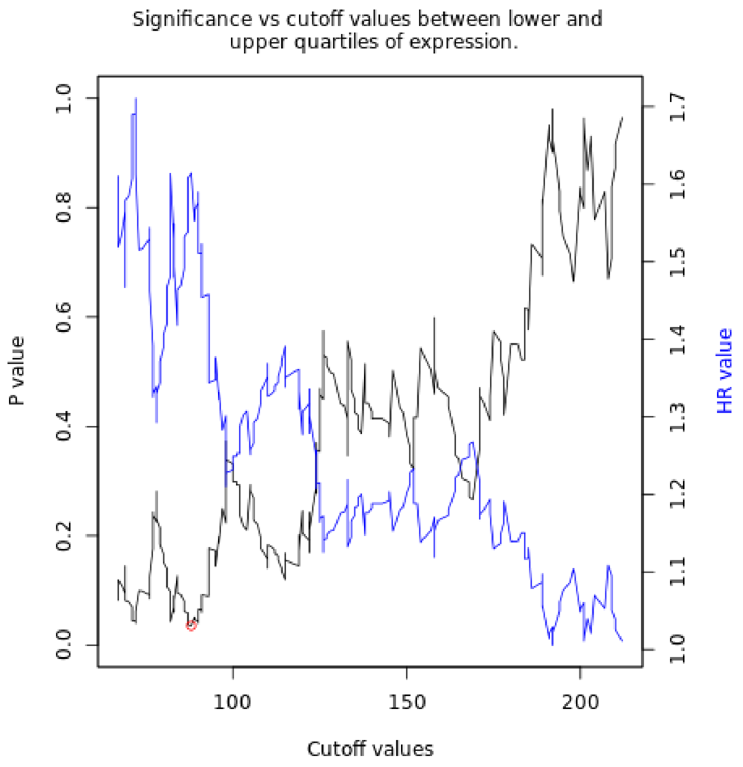

Download p values vs. cutoff table

Upper quartile survival

Low expression cohort (months)	High expression cohort (months)
108.37	49.47

Click here for a permanent link

Restart

You can save the plots by right-clicking the image and then selecting "Save image as...". To generate a high resolution TIFF image, please adjust the "Settings" in the analysis page.

KMplot.com Copyright © 2009-2022 | Contact | Sitemap
